# Critical role of RanBP2-mediated SUMOylation of Small Heterodimer Partner in maintaining bile acid homeostasis

**DOI:** 10.1038/ncomms12179

**Published:** 2016-07-14

**Authors:** Dong-Hyun Kim, Sanghoon Kwon, Sangwon Byun, Zhen Xiao, Sean Park, Shwu-Yuan Wu, Cheng-Ming Chiang, Byron Kemper, Jongsook Kim Kemper

**Affiliations:** 1 Department of Molecular and Integrative Physiology, University of Illinois at Urbana-Champaign, Urbana, Illinois 61801, USA; 2 Laboratory of Proteomics and Analytical Technologies, Advanced Technology Program, SAIC-Frederick, Inc., Frederick National Laboratory for Cancer Research, Frederick, Maryland 21702, USA; 3 Simmons Comprehensive Cancer Center, Department of Biochemistry, and Department of Pharmacology, University of Texas, Southwestern Medical Center, Dallas, Texas 75390, USA

## Abstract

Bile acids (BAs) are recently recognized signalling molecules that profoundly affect metabolism. Because of detergent-like toxicity, BA levels must be tightly regulated. An orphan nuclear receptor, Small Heterodimer Partner (SHP), plays a key role in this regulation, but how SHP senses the BA signal for feedback transcriptional responses is not clearly understood. We show an unexpected function of a nucleoporin, RanBP2, in maintaining BA homoeostasis through SUMOylation of SHP. Upon BA signalling, RanBP2 co-localizes with SHP at the nuclear envelope region and mediates SUMO2 modification at K68, which facilitates nuclear transport of SHP and its interaction with repressive histone modifiers to inhibit BA synthetic genes. Mice expressing a SUMO-defective K68R SHP mutant have increased liver BA levels, and upon BA- or drug-induced biliary insults, these mice exhibit exacerbated cholestatic pathologies. These results demonstrate a function of RanBP2-mediated SUMOylation of SHP in maintaining BA homoeostasis and protecting from the BA hepatotoxicity.

## Abstract (web summary)

As signalling molecules, bile acids (BAs) can affect metabolism, but due to detergent-like properties, BA levels must be tightly regulated. Here, Kim *et al.* show that RanBP2, a nucleoporin, maintains BA homoeostasis through SUMOylation of Small Heterodimer Partner (SHP), an orphan nuclear receptor.

## Graphical abstract

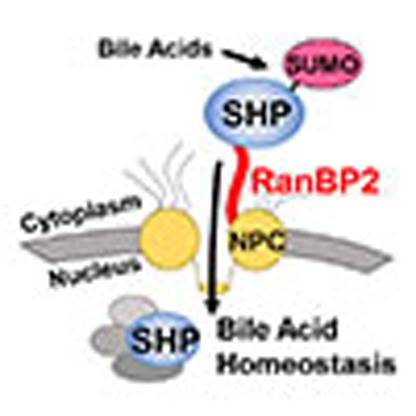

Bile acids (BAs) have traditionally been considered to have a simple dietary role for the absorption of lipid-soluble nutrients, but increasing evidence demonstrates that BAs are also signalling molecules that profoundly affect metabolism and energy balance^1^,^2^,^3^. Because of the detergent-like toxic properties of excess BAs, their levels must be tightly controlled through feedback transcriptional regulation of BA synthesis, transport and metabolism. Deficiencies in these homoeostatic responses result in abnormal accumulation of BAs in the liver, resulting in cholestatic diseases and liver injury^4^,^5^,^6^.

The orphan nuclear receptor, Small Heterodimer Partner (SHP, NR0B2), acts as a co-repressor of many transcription factors and plays a key role in maintaining BA homeostasis^7^,^8^. In response to increased hepatic BA levels, SHP inhibits expression of the BA synthetic genes, *Cyp7a1 and Cyp8b1* (ref. 7), and the BA import transporter gene, *Ntcp*^9^, by recruiting repressive histone-modifying enzymes, such as HDACs and LSD1 (refs 10, 11). An endogenous ligand for SHP has not been identified, but its gene repression function and protein levels are modulated by BA signal-regulated post-translational modifications, such as, phosphorylation and ubiquitination^11^,^12^.

Ran-binding protein 2 (RanBP2/Nup358) is the largest component of cytoplasmic filaments that emanate from the nuclear pore complex and is a small ubiquitin-related modifier (SUMO) ligase^13^,^14^. Functions for RanBP2 in bidirectional transport of proteins and RNA between the nucleus and cytoplasm and in mitotic spindle organization during the cell cycle have been reported in cultured cells^15^. The *in vivo* role of RanBP2, however, remains largely unknown. Mice lacking RanBP2 were embryonic lethal, although heterozygous mice had reduced cell death upon oxidative stress and impaired glucose metabolism in the retina^16^. A few proteins, including RanGAP, HDAC4 and Borealin, have been identified as targets of RanBP2 SUMOylation^13^,^14^,^17^, but physiological roles for RanBP2-mediated SUMOylation of these proteins are not known.

Here, we show an unexpected function of RanBP2 in maintaining BA homoeostasis through SUMOylation of SHP. Upon BA signalling, RanBP2 SUMOylates SHP at K68, which is required for nuclear transport and the gene repression function of SHP in feedback inhibition of BA biosynthesis that is critical for maintaining BA homoeostasis and protecting against liver toxicity.

## Results

### RanBP2 is a novel SHP-interacting protein

**Figure 1:**
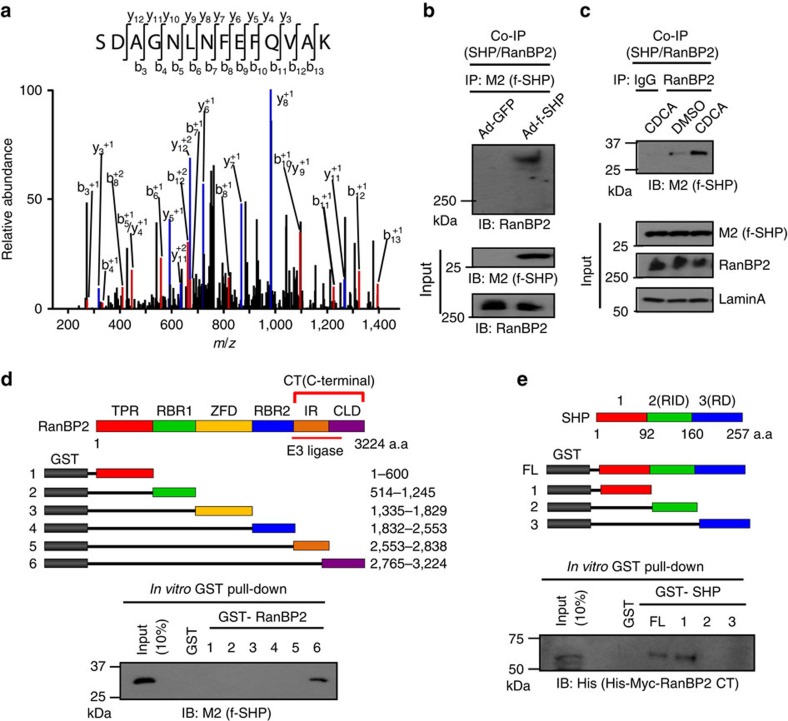
RanBP2 is a novel SHP-interacting protein. (**a**) Liquid chromatography–mass spectrometry spectrum identifying a RanBP2 peptide in a flag-SHP immunoprecipitate. (**b**,**c**) Flag-SHP expressed in HepG2 cells (**b**) or in primary mouse hepatocytes that were treated with vehicle (dimethylsulphoxide) or 50 μM CDCA for 30 min (**c**) was precipitated by M2 antibody and RanBP2 was detected in the immunoprecipitates by IB. (**d**) A schematic representation of the fragments of RanBP2 that were fused to GST (top). GST fusion proteins were incubated with flag-SHP protein and binding of flag-SHP to the GST-proteins was detected by IB with M2 antibody. CLD, cyclophilin-like domain; CT, C-terminal; IR, internal repeat; RBR1, RAN-binding domain1; RBR2, RAN-binding domain2; TPR, tetratricopeptide repeat; ZFD, zinc-finger motif domain. (**e**) A schematic representation of the fragments of SHP that were fused to GST (top). GST fusion proteins were incubated with His-Myc-tagged RanBP2 CT (C-terminal) protein and binding of His-Myc-RanBP2 CT to the GST-proteins was detected by IB with His antibody. RD, repression domain; RID, receptor interaction domain.

In proteomic analyses of flag-SHP complexes from HepG2 cells treated with vehicle or a primary BA, chenodeoxycholic acid (CDCA), RanBP2, a SUMO E3 ligase and the largest component of cytoplasmic filaments of nuclear pore complexes, was unexpectedly detected in CDCA-treated samples (Fig. 1a). The RanBP2-SHP interaction was confirmed by co-immunoprecipitation (CoIP) (Fig. 1b), and treatment with CDCA increased the interaction in primary mouse hepatocytes (Fig. 1c). In glutathione *S*-transferase (GST) pull-down assays, RanBP2 interacted with SHP through its C-terminal region, the cyclophilin-like domain, which overlaps with its E3 ligase domain (Fig. 1d), and SHP bound to RanBP2 through its N-terminal region (Fig. 1e and Supplementary Fig. 1). These biochemical studies suggest that RanBP2 directly interacts with SHP and the interaction is increased by BA treatment.

### RanBP2 mediates SUMO2 modification of SHP at Lys-68

**Figure 2:**
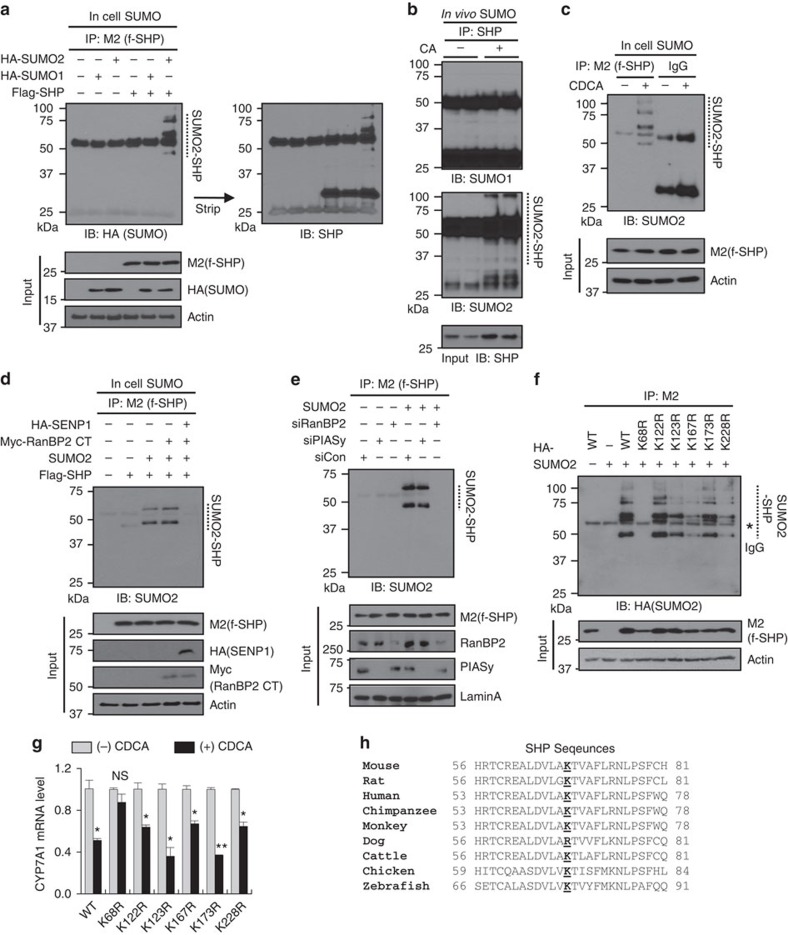
SHP is SUMOylated at K68 by RanBP2. (**a**,**c**–**f**) HepG2 cells were transfected with expression plasmids or siRNA as indicated. Cells were treated with CDCA for 15 min, flag-SHP was immunoprecipitated and SUMOylated SHP in the immunoprecipitates was detected by IB with SUMO or HA antibody. Input levels of the proteins were detected by IB as indicated. (**b**) *In vivo* SUMO assay: SUMO1- or SUMO2-levels of endogenous SHP in livers of mice fed normal or CA chow for 3 h were detected by IP/IB. (**e**) Flag-SHP WT and mutants with Lys to Arg mutations were expressed with SUMO2 in HepG2 cells. The flag-tagged proteins were immunoprecipitated and SUMOylated SHP was detected by IB with HA antibody. (**g**) Flag-SHP or the indicated mutants were expressed in HepG2 cells that were treated with vehicle or 50 μM CDCA for 6 h, and levels of *CYP7A1* mRNA were determined. The control samples are set to 1 for each SHP protein. Values represent mean±s.e.m. Statistical significance was determined by the Student's *t*-test, (*n*=3, *P<0.05, **P<0.005 and NS, statistically not significant). (**h**) Comparison of amino acid sequences adjacent with K68 in the indicated vertebrate species.

Since SHP interacts with a SUMO ligase, RanBP2, we next examined if SHP is a target of RanBP2-mediated SUMOylation. SUMOylation of SHP was detected in CDCA-treated HepG2 cells expressing SUMO2, but not SUMO1 (Fig. 2a), suggesting that SHP is a target of SUMO2 modification. Consistent with these results, cholic acid (CA) feeding markedly increased SUMO2-SHP levels, but not SUMO1-SHP levels, in mouse liver extracts (Fig. 2b). In cell SUMO assays, SUMO2-modified SHP levels were increased by CDCA treatment (Fig. 2c) and overexpression of SENP1, which removes the SUMO modification, decreased the SUMO2- SHP levels (Fig. 2d). Importantly, downregulation of endogenous RanBP2 completely blocked SHP SUMOylation, whereas downregulation of PIASy, another E3 SUMO ligase, did not (Fig. 2e). Incubation of a catalytically active RanBP2 fragment^13^,^14^ resulted in SUMO2 modification of SHP *in vitro* (Supplementary Fig. 2). These results indicate that RanBP2 mediates SUMO2 modification of SHP.

The consensus motif for SUMOylation at Lys, ψKxD/E (refs 15, 18) is not present in SHP, but SUMOylation can occur at non-consensus SUMO motifs^19^. We, therefore, mutated each of the six Lys residues in SHP to Arg (R). In in-cell SUMO assays, SUMOylation was completely blocked only by the K68R mutant (Fig. 2f). SUMOylation of wild-type (WT) SHP was substantially reduced by the K68R mutation *in vitro* (Supplementary Fig. 3a). In CDCA-treated HepG2 cells, only mutation of Lys-68 eliminated the statistically significant inhibition by SHP of expression of BA synthetic genes, *CYP7A1* (Fig. 2g) and *CYP8B1* (Supplementary Fig. 3b). Notably, Lys-68 is highly conserved among vertebrate species (Fig. 2h), suggesting its functional importance. These results from biochemical studies, taken together, indicate that RanBP2 mediates SUMOylation of mouse SHP, mainly at K68 (K65 in human), upon BA treatment.

### SUMOylation of SHP facilitates its nuclear localization

**Figure 3:**
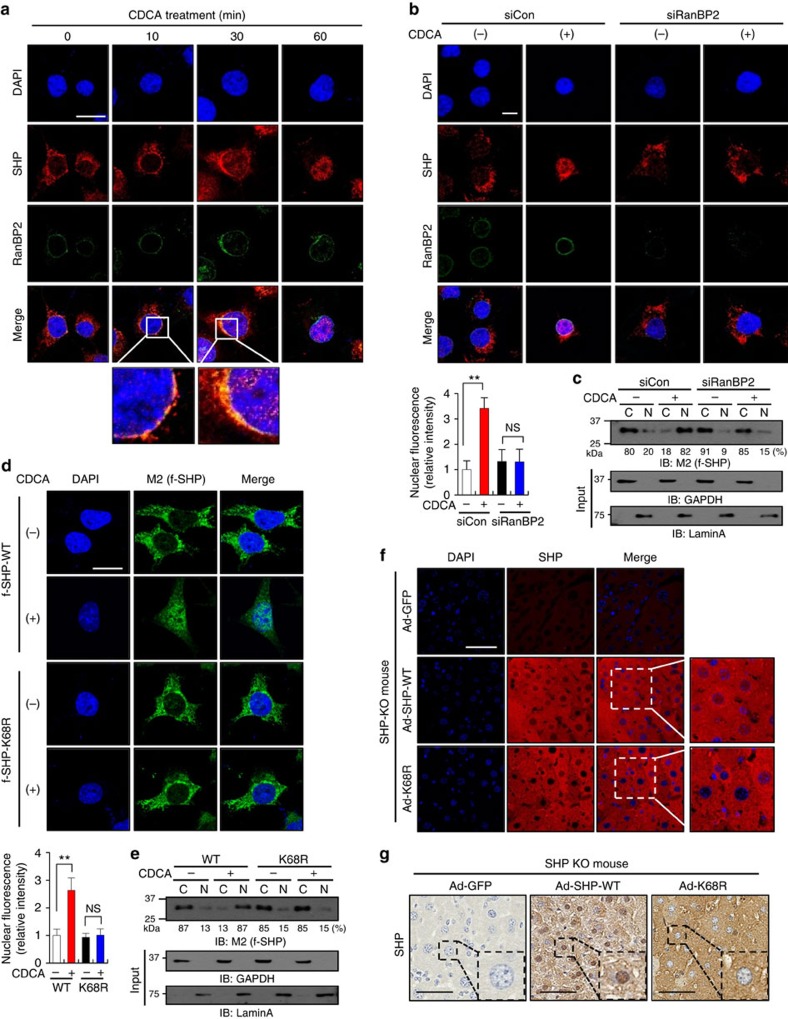
RanBP2-mediated SUMOylation at K68 facilitates nuclear localization of SHP. (**a**–**c**) Hepa1c1c7 cells were incubated with serum-free media for 24 h and then, treated with 50 μM CDCA for the indicated times (**a**) (scale bar, 20 μm) or were incubated with siRNA for RanBP2 or control siRNA for 48 h and then treated for 60 min with 50 μM CDCA (**b**) (scale bar, 10 μm). Endogenous RanBP2 and SHP were detected by IF using confocal laser scanning and the intensity of red fluorescence (SHP) in the nucleus was quantified (lower left). Values represent mean±s.e.m. Statistical significance was determined by the Student's *t*-test, (*n*=20 cells, **P<0.005 and NS, statistically not significant). (**c**) Hepa1c1c7 cells expressing flag-SHP were treated with siRNA as indicated and cell extracts were fractionated into cytoplasmic (C) and nuclear (N) fractions, and flag-SHP was detected by IB with M2 antibody. The intensities of the bands were quantified and the percentage of the total in the nuclear and cytoplasmic fractions for each sample is shown below the blot. The cytoplasmic marker, GAPDH, and nuclear marker, lamin A, were detected by IB. (**d**) Flag-SHP or the K68R mutant was expressed in Hepa1c1c7 cells that were treated with vehicle or 50 μM CDCA for 60 min. Flag-SHP was detected with M2 antibody using IF and the intensity of green fluorescence (SHP) in the nucleus was quantified (lower left). Scale bar, 20 μm. Values represent mean±s.e.m. Statistical significance was determined by the Student's *t*-test, (*n*=20 cells, **P<0.005 and NS, statistically not significant). (**e**) Hepa1c1c7 cells expressing flag-SHP WT or the K68R mutant were treated with CDCA, and flag-SHP in cytoplasmic (C) and nuclear (N) fractions was detected by IB. The intensities of the bands were quantified and the percentage of the total in the nuclear and cytoplasmic fractions for each sample is shown below the blot. (**f**,**g**) Flag-SHP WT or the K68R mutant was adenovirally expressed in SHP-KO mice and detected by IF (**f**) or IHC (**g**) as described in Methods. Scale bar, 50 μm (**f**,**g**).

We then examined the effect of RanBP2-mediated SUMOylation of SHP on subcellular localization of SHP. Since RanBP2 is component of cytoplasmic filaments emanating from the nucleopore complex^13^,^15^, we first examined if SHP is co-localized with RanBP2. In vehicle-treated Hepa1c1c7 cells, endogenous SHP was detected predominantly in the cytosol (Fig. 3a). Interestingly, 10 min after CDCA treatment, SHP was concentrated at the nuclear envelope region, co-localizing with RanBP2; at 30 min, co-localization was still evident with increased nuclear SHP levels; and at 60 min, SHP was predominantly nuclear. RanBP2 protein levels detected by immunoblotting (IB) were transiently increased about twofold by CDCA treatment for 30 min, but the mRNA levels of RanBP2 were not changed (Supplementary Fig. 4). These results suggest that, upon BA treatment, RanBP2 and SHP transiently interact at the nuclear pore region, before nuclear translocation of SHP.

To further determine if the increased nuclear localization of SHP after CDCA treatment is dependent on RanBP2, endogenous RanBP2 was downregulated by siRNA. The increased nuclear level of SHP after CDCA treatment for 60 min was completely blocked when RanBP2 was downregulated (Fig. 3b), which was also observed biochemically in cellular fractionation studies (Fig. 3c). Further, the increase in nuclear localization of SHP after CDCA treatment was attenuated by the K68R mutation (Fig. 3d,e). These findings suggest that SUMOylation of SHP at K68 by RanBP2 is required for BA signal-induced nuclear transport of SHP.

We further examined the effect of SUMOylation of SHP on subcellular localization of SHP *in vivo*. Endogenous SHP in liver was detected in both cytoplasm and nucleus, mostly in the cytoplasm, in fasted mice, and the nuclear abundance of SHP was increased upon feeding or more markedly increased by feeding CA chow (Supplementary Fig. 5). Using adenoviral infection, SHP-WT or the SUMO-defective K68R mutant was expressed in SHP-knockout (KO) mice at levels similar to those detected in CA-fed mice (Supplementary Fig. 6a). The nuclear abundance of SHP detected by immunofluorescence (IF) (Fig. 3f) or immunohistochemistry (IHC; Fig. 3g) was substantially diminished by expression of the K68R mutant compared with SHP WT in these mice. These subcellular localization studies in hepatic cells and in mice *in vivo* clearly demonstrate that RanBP2-mediated SUMOylation of SHP at K68 facilitates its nuclear translocation.

### SUMOylated SHP is required for its repression function

**Figure 4:**
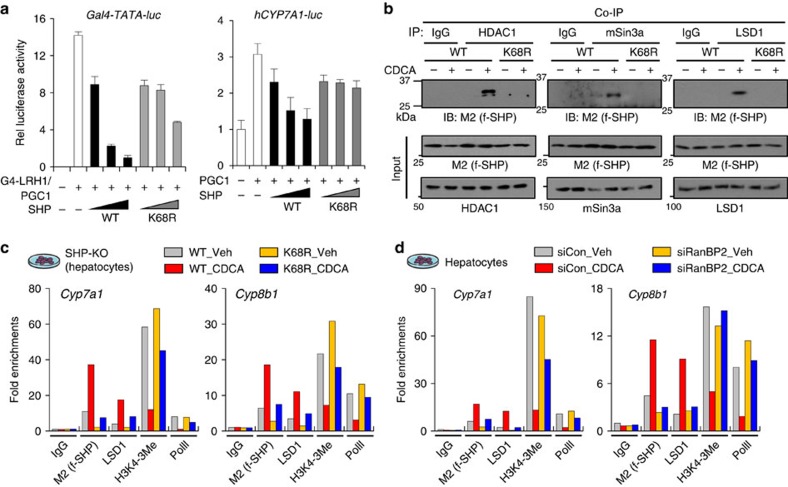
SUMOylation at K68 increases SHP-mediated epigenetic repression of BA synthetic genes. (**a**) Luciferase reporter assay: HepG2 cells were transfected with plasmids as indicated and luciferase activities were normalized to β-galactosidase activity (*n*=3). (**b**) CoIP: Flag-SHP WT or the K68R mutant was expressed in HepG2 cells and then, the cells were treated with 50 μM CDCA for 30 min. LSD1, HDAC1 or mSin3A was immunoprecipitated with their respective antibodies and flag-SHP in the immunoprecipitates was detected by M2 antibody. (**c**,**d**) ChIP: (**c**) Ad-flag-SHP or the K68R mutant were expressed in SHP-KO primary mouse hepatocytes or (**d**) endogenous RanBP2 was downregulated by siRNA in normal mouse hepatocytes and then, cells were treated with vehicle or 50 μM CDCA for 1 h. Chromatin was isolated and precipitated with IgG or antibody as indicated and the enrichment of DNA sequence for *Cyp7a1 or Cyp8b1* was determined.

LRH-1 is an *in vivo* activator of BA synthetic genes, *Cyp7a1 and Cyp8b1,* and a well-known SHP-interacting nuclear receptor^20^,^21^,^22^,^23^. In luciferase reporter assays, SHP-mediated repression of LRH-1 and *CYP7A1* transactivation was markedly reversed by the K68R mutation (Fig. 4a). These results suggest that SUMOylation of SHP at K68 is important for inhibition of LRH-1 or *CYP7A1* activity.

SHP inhibits expression of BA synthetic genes by recruiting repressive histone-modifying enzymes, like HDACs and LSD1 (refs 10, 24, 25). In CoIP studies, SHP interaction with a co-repressor mSin3A and repressive histone-modifying enzymes, LSD1 and HDAC1, was abolished by the K68R mutation (Fig. 4b). In chromatin immunoprecipitation (ChIP) assays, BA treatment increased occupancy of SHP and LSD1 and decreased H3K4-me3 levels and RNA pol II occupancy at *Cyp7a1 and Cyp8b1* in hepatocytes expressing SHP-WT, but these effects were attenuated by the K68R mutation (Fig. 4c). Similar effects were observed with downregulation of RanBP2 (Fig. 4d). Further, in mouse liver, acute (6 h) CA feeding increased the occupancy of SHP and SUMO2 and decreased occupancy of RNA polymerase II at *Cyp7a1 and Cyp8b1*, and a BA import transporter, *Ntcp* (Supplementary Fig. 7). These results suggest that SUMOylation of SHP at K68 is also important for its gene repression functions by increasing the interaction with repressive histone-modifying enzymes.

### Correlation between phospho- and SUMO2-SHP

**Figure 5:**
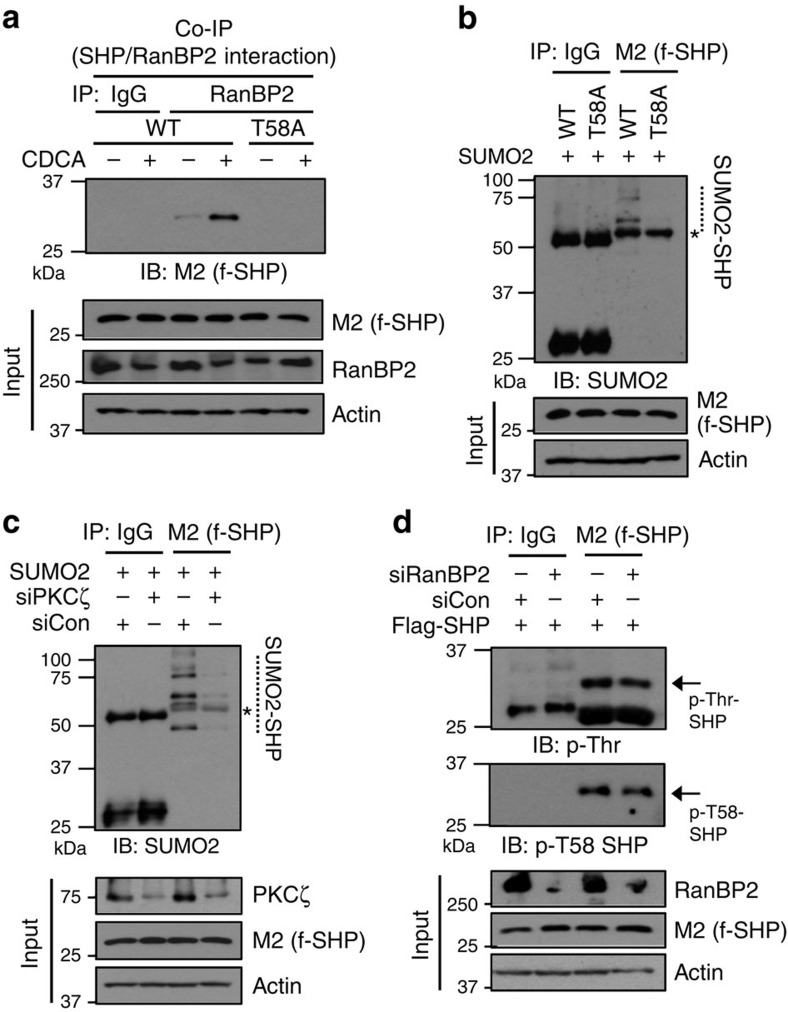
Phosphorylation of SHP at Thr-58 is likely important for RanBP2-mediated SUMOylation of SHP. HepG2 cells were transfected with expression plasmids or siRNA for RanBP2 or PKCζ as indicated, and then, the cells were treated with 50 μM CDCA for 15 min. (**a**) SHP interaction with RanBP2 was detected by CoIP. (**b**–**d**) Phosphorylated mouse SHP at T58 (T55 in human SHP) or SUMOylated SHP at K68 was detected by the IP/IB method or phosphorylated SHP at T58 was detected by IB using the P-T58 SHP antibody. Protein levels in input samples were detected by IB.

An important question is how BA signalling increases RanBP2-mediated SUMOylation of SHP. Since mouse SHP is phosphorylated at Thr-58 (Thr-55 in human SHP) by PKCζ upon BA or FGF15 signalling^11^, we examined potential cross-talk between the phosphorylation and SUMOylation of SHP. The BA-induced RanBP2 interaction with SHP was completely abolished in the phosphorylation-defective T58A mutant (Fig. 5a). Further, the T58A SHP mutant was not SUMOylated (Fig. 5b) and downregulation of PKC∂ζ abolished SUMOylation of SHP (Fig. 5c), while downregulation of RanBP2 did not affect phosphorylation of SHP at Thr-58 (Fig. 5d). These results suggest that phosphorylation of SHP by PKCζ is upstream of RanBP2-mediated SUMOylation.

Since nuclear receptors, FXR and LRH-1, are key nuclear receptors that control BA levels and both are targets of SUMO modifications^26^,^27^,^28^, we further examined the relevance of the BA signal-PKCζ-RanBP2 pathway in SUMOylation of these proteins. Downregulation of PKCζ or RanBP2 did not change the levels of SUMOylated FXR and LRH-1 in CDCA-treated HepG2 cells (Supplementary Fig. 8). Consistent with our recent finding that PIASy mediates SUMO2 modification of FXR^26^, downregulation of PIASy completely abolished SUMO2-FXR levels (Supplementary Fig. 8). Further, CDCA treatment did not induce interaction of RanBP2 with FXR or LRH-1, while it substantially increased RanBP2 interaction with SHP (Supplementary Fig. 9). These results suggest that the PKCζ-RanBP2 pathway exhibits specificity for SHP with respect to these two nuclear receptors, but do not eliminate a role for this pathway for other transcriptional regulators. These results suggest that the BA signal-induced phosphorylation of SHP at Thr-58 (Thr-55 in human) by PKCζ is important for RanBP2-mediated SUMOylation of SHP.

### Role of SUMOylation of SHP in regulating liver BA levels

**Figure 6:**
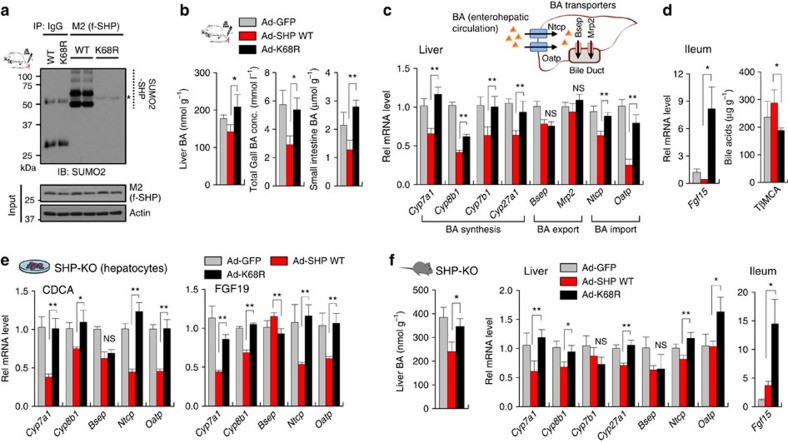
Effects of SUMOylation of SHP on BA regulation in mice without biliary insults. (**a**–**d**) Mice were tail vein injected with Ad-GFP, SHP WT or K68R mutant, and 3 weeks later, serum and tissues were collected. (**a**) *In vivo* SUMO assay: Flag-tagged SHP in liver extracts was immunoprecipitated with M2 antibody. SUMOylated SHP in the immunoprecipitates was detected by IB with SUMO2 antibody. (**b**) The levels of BAs in the liver, gall bladder or intestine were determined and values are expressed per gram tissue weight. (**c**) quantitative qRT-PC: Liver BA transporters are illustrated (top). The mRNA levels of the indicated genes in the liver were measured by qRT-PCR. (**d**) The mRNA levels of intestinal *Fgf15* and TβMCA levels in the intestine. (**e**) WT-SHP or the K68R mutant was expressed in hepatocytes isolated from SHP-KO mice, and expression of indicated genes was measured by qRT-PCR. (**f**) SHP-WT or the K68R mutant was adenovirally expressed in SHP-KO mice and liver BA levels, and expression of indicated genes were measured. The values for WT SHP are set to 1. Values represent mean±s.e.m. Statistical significance was determined by the Student's *t*-test, (*n*=5 mice, *P<0.05, **P<0.005, and NS, statistically not significant). qRT-PCR, quantitative real-time PCR.

To examine the *in vivo* role of SHP SUMOylation, SHP-WT or the SUMO-defective K68R mutant was adenovirally expressed in mouse liver at levels similar to those detected in CA-fed mice (Supplementary Fig. 6b). SUMO2-modified SHP levels in liver extracts were markedly decreased in mice expressing the K68R mutant (Fig. 6a), confirming that Lys-68 is the major SUMO site in SHP *in vivo*. BA levels in the liver, gall bladder and small intestine were significantly increased in mice expressing the K68R-SHP mutant compared with the WT (Fig. 6b). The decreased expression of the BA synthetic genes, including *Cyp7a1*, and BA import transporters, *Ntcp*^9^, and *Oatp*, observed after expression of SHP WT was reversed by the K68R mutation, whereas no effect of the mutation was observed for the BA export transporters, *Bsep and Mrp2* (Fig. 6c). These transcriptional outcomes in mice expressing the SHP K68R mutant that lead to increased BA synthesis and increased BA import are consistent with increased liver BA levels in these mice. These results taken together indicate that under physiological conditions without biliary insults, SUMO2 modification at K68 in SHP plays an important role in transcriptional responses to effectively regulate hepatic BA levels.

The liver and gut communicate with each other in regulation of hepatic BA synthesis through an intestinal hormone, FGF15 (FGF15 in mice; FGF19 in human)^29^. Notably, intestinal expression of *Fgf15,* a strong inhibitor of BA synthetic genes, particularly *Cyp7a1*, that is induced by the BA nuclear receptor FXR (ref. 30), was increased in mice expressing the K68R-SHP mutant compared with WT (Fig. 6d). We also examined the BA composition in the liver and intestine in these mice. In contrast, the liver, the intestinal BA composition was altered by the K68R-SHP mutation, including decreased levels of an antagonist for FXR, TβMCA (ref. 29) (Fig. 6d and Supplementary Fig. 10). Decreased TβMCA levels in these mice expressing K68R may contribute to the increased expression of *Fgf15*. Despite increased intestinal *Fgf15* expression, hepatic expression of *Cyp7a1* and *Cyp8b1* was significantly increased (Fig. 6c), suggesting that SUMOylation of SHP plays an important role in mediating FGF15 inhibition of BA synthetic genes. Supporting this idea, treatment with FGF19, as well as CDCA, decreased expression of *Cyp7a1* and *Cyp8b1* in hepatocytes from SHP-KO mice expressing SHP-WT, but the FGF19-mediated inhibition of these genes was blunted with the K68R mutation (Fig. 6e).

To avoid confounding effects due to endogenous SHP in *in vivo* studies, we expressed physiological levels of SHP-WT or the SUMO-defective K68R mutant in livers of SHP-KO mice (Supplementary Fig. 6a). Compared with the results from normal mice (Fig. 6b,c), similar effects on liver BA levels and expression of BA synthetic and transporter genes and *Fgf15* were observed in SHP-KO mice expressing the K68R mutant (Fig. 6f). These results, taken together, indicate that SUMOylation of SHP at K68 plays an important role in regulating liver BA levels under physiological conditions.

### Effects of downregulation of RanBP2 in mice

**Figure 7:**
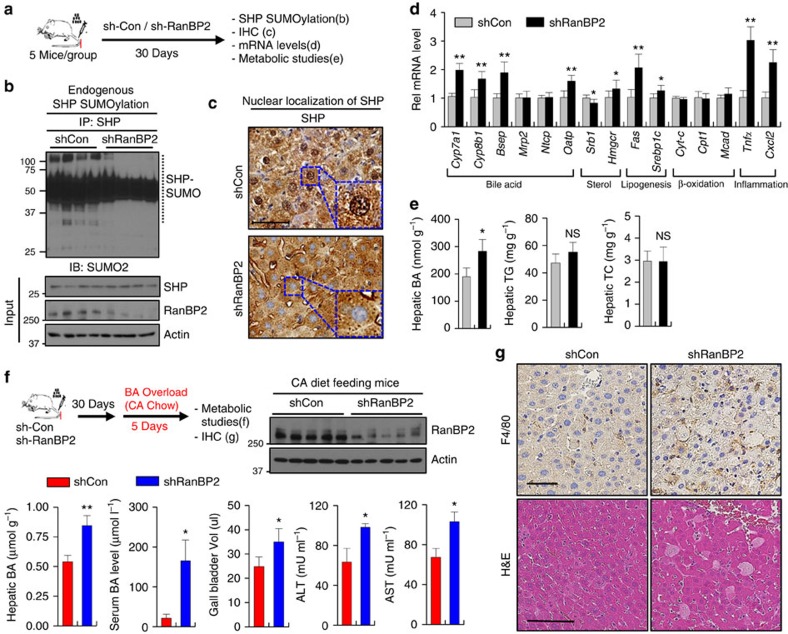
Effects of downregulation of RanBP2 in BA regulation in mice. (**a**–**d**) Experiments without biliary insults. (**a**) Experimental Outline. (**b**) *In vivo* SUMO assay: SUMO-SHP levels in RanBP2-downregulated mice were detected by IP/IB. Endogenous SHP in liver extracts was immunoprecipitated with SHP antibody and SUMOylated SHP in the immunoprecipitates was detected by IB using SUMO2 antibody. Levels of RanBP2 or SHP in liver extracts after expression of control or RanBP2 shRNA were detected by IB. (**c**) Effects of downregulation of RanBP2 on subcellular localization of SHP in the mouse liver. SHP was detected by IHC. Scale bar, 50 μm. Effects on expression of the indicated genes (**d**) or the levels of BA, TG, and cholesterol (**e**) in the liver. In **e**, values for the control shRNA groups are set to 1. (**f**,**g**) Experiments with a biliary insult by BA overload. (**f**) Experimental outline and protein levels of RanBP2 detected by IB (top). The volume of the gall bladder, serum BA levels, liver BA levels, and serum ALT and AST levels were determined. BA levels are expressed per g of liver wet weight. Values represent mean±s.e.m. Statistical significance was determined by the Student's *t*-test, (*n*=5 mice, *P<0.05, **P<0.005, and NS, statistically not significant) (**d**–**f**). (**g**) Liver sections were stained with haematoxylin and eosin (H&E) to detect necrosis and macrophages were detected (brown) with F4/80 antibody. Scale bar, 100 μm.

To investigate the *in vivo* role of RanBP2-mediated SUMOylation of SHP, we examined the effects of downregulation of RanBP2 on SUMOylation and nuclear localization of SHP and BA regulation. Since RanBP2-KO mice are embryonic lethal^16^, RanBP2 was downregulated in mice by lentiviral-mediated expression of RanBP2 shRNA (Fig. 7a). Importantly, downregulation of RanBP2 resulted in markedly decreased levels of endogenous SUMO2-SHP (Fig. 7b), but not SUMO1-SHP levels (Supplementary Fig. 11) in the liver, and substantially decreased nuclear levels of SHP (Fig. 7c). Further, the downregulation of RanBP2 resulted in increased expression of BA metabolic, lipogenic and inflammatory genes (Fig. 7d), and liver BA levels were increased, although liver triglyceride and total cholesterol levels were not significantly changed (Fig. 7e). Increased liver BA levels cause liver injury and inflammation^6^,^31^, which likely underlies the increased expression of the inflammatory genes, *Tnfα* and *Cxcl2* (Fig. 7d).

When these RanBP2-downregulated mice were challenged by chronic BA feeding (Fig. 7f, top), liver BA levels were elevated ∼50%, and the gall bladder volume was significantly increased compared with control mice (Fig. 7f). In contrast to untreated mice (Fig. 7d), expression of *Cyp7a1* was decreased by downregulation of RanBP2 in the BA-challenged mice (not shown). Strikingly, serum BA levels were substantially increased, as were the liver toxicity markers, serum ALT and AST levels, in these mice (Fig. 7f). Further, pathological changes in the liver were evident including loosened cellular structure, degeneration of hepatocytes, increased liver necrotic area and increased macrophage infiltration (Fig. 7g). These results indicate that downregulation of RanBP2 in mice decreased levels of endogenous SUMO2-SHP and also increased hepatic BA levels and liver toxicity, suggesting a functional role for RanBP2 in BA regulation and protection against hepatic toxicity.

### The SHP K68R mutation exacerbates cholestatic symptoms

**Figure 8:**
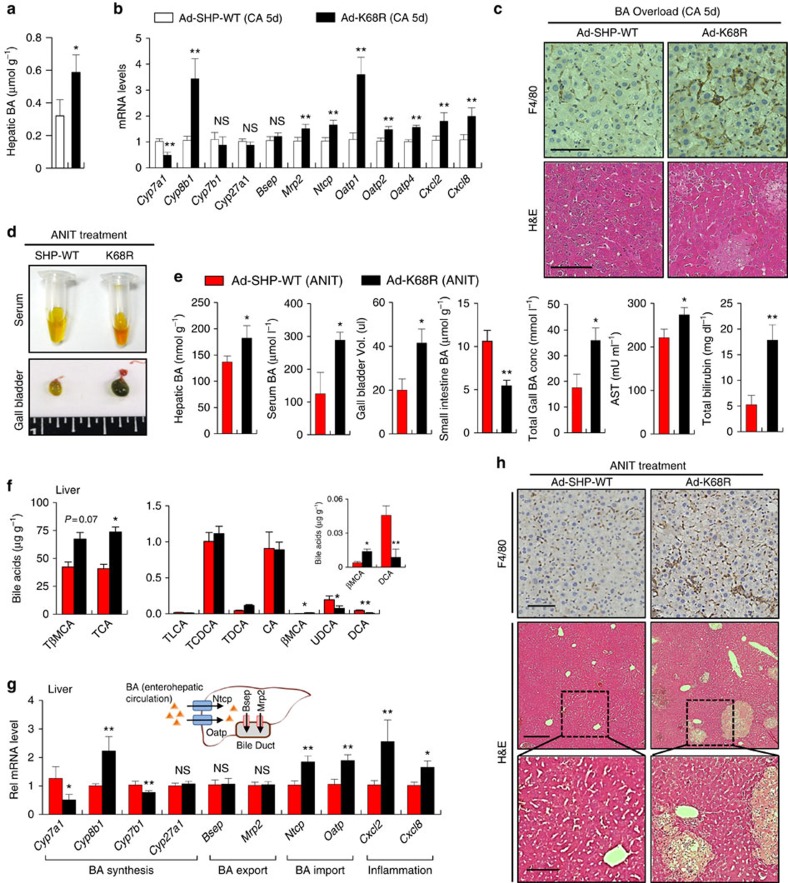
Mice expressing the K68R SHP mutant have exacerbated cholestatic symptoms upon biliary insults. (**a**–**c**) BA overload experiments. (**a**) Mice were fed 0.5% CA chow for 5 days and hepatic and serum BA levels were determined. Values represent mean±s.e.m. (**b**) The mRNA levels of the indicated hepatic genes were measured. Values represent mean±s.e.m. Statistical significance was determined by the Student's *t*-test, (*n*=5 mice, *P<0.05, **P<0.005, and NS, statistically not significant) (**a**,**b**). (**c**) Liver sections were stained and macrophages detected as in Fig. 7g. Scale bar, 100 μm. (**d**–**h**) ANIT experiments. (**d**) Pictures of serum and gall bladders from control and ANIT-treated mice expressing SHP or the K68R mutant. (**e**) Hepatic, serum, intestinal and gall bladder BA levels, serum AST levels and total bilirubin levels. Values represent mean±s.e.m. (**f**) BA composition in the liver. Values represent mean±s.e.m. (**g**) The mRNA levels of the indicated genes in liver and intestine were measured. Values represent mean±s.e.m. Statistical significance was determined by the Student's *t*-test, (*n*=5 mice, *P<0.05, **P<0.005 and NS, statistically not significant) (**e**–**g**). (**h**) Liver sections were stained and macrophages detected as in Fig. 7g. Scale bar, 50 μm (F4/80), 250 μm and 100 μm (H&E).

To further determine the functional role of RanBP2-mediated SUMOylation of SHP, we challenged mice expressing SHP-WT or the SUMO-defective K68R mutant by either chronic BA feeding or by a more potent biliary insult, treatment with α-naphthyl isothiocyanate (ANIT), which induces intrahepatic cholestasis by injuring biliary epithelial cells^31^,^32^. In mice fed CA-supplemented chow for 5 days, liver BA levels were significantly increased nearly twofold in mice expressing the K68R mutant compared with the WT (Fig. 8a). Further, while mRNA levels of *Cyp7a1* was decreased, mRNA levels of the BA synthetic gene, *Cyp8b1*, BA import genes, and inflammatory genes were increased (Fig. 8b), and pathological changes in the liver were evident with increased liver necrosis and infiltration of macrophages (Fig. 8c). These results indicate that increased BA levels and liver toxicity in response to chronic BA overload occurred in mice expressing the K68R mutant compared with SHP-WT.

In ANIT-treated mice, serum colour was more darkly yellow and the gall bladder was enlarged in the mice expressing the K68R mutant compared with WT-SHP (Fig. 8d). Liver and serum BA levels were substantially increased; bile volume in the gall bladder and total BA levels were increased nearly twofold, while intestinal BA levels were reduced; and serum AST and total bilirubin levels were also significantly increased (Fig. 8e). The BA composition in the liver also markedly altered such that the levels of taurine-conjugated BAs trended higher, with decreased hepatic levels of UDCA, an effective BA for treatment for cholestatic liver disease^33^, in mice expressing the K68R-SHP mutant (Fig. 8f).

The mRNA levels of *Cyp8b1,* which regulates BA hydrophobicity^2^,^3^, were highly increased, while those of *Cyp7a1 and Cyp7b1* were decreased in the mice expressing the K68R mutant (Fig. 8g). Expression of the BA import genes, *Ntcp and Oatp*, and inflammatory genes was significantly increased, while that of the BA export genes, *Bsep and Mrp2,* was unchanged. Gene expression patterns in the ileum were also altered in mice expressing the K68R mutant with decreased expression of *Fgf15* and increased expression of BA transporters, *Ostα and Ostβ* (Supplementary Fig. 12). These altered transcriptional and metabolic outcomes should increase liver BA levels and cholestatic symptoms. Consistent with these detrimental outcomes, pathological changes in the liver, including liver necrosis, and increased inflammation were evident in mice expressing the K68R mice compared with mice expressing SHP-WT upon ANIT treatment (Fig. 8h). These *in vivo* studies from chronic BA feeding or ANIT experiments show that the response to biliary insults was impaired in mice expressing the SUMO-defective K68R SHP mutant, resulting in exacerbated cholestatic symptoms.

## Discussion

**Figure 9:**
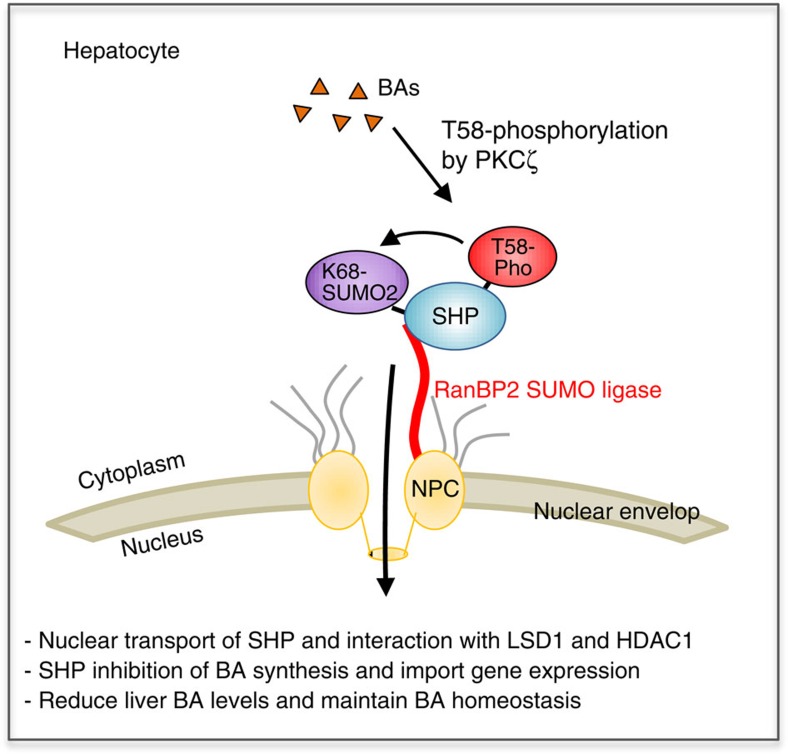
Model. In response to elevated hepatic BA levels, interaction between RanBP2 and SHP is increased. RanBP2, then, mediates SUMO2 modification of SHP at K68, which facilitates the nuclear transport of SHP and increased SHP interaction with histone-modifying enzymes, LSD1 and HDAC1, resulting in feedback repression of BA synthesis and import transport that is critical for reducing liver BA levels and maintaining BA homoeostasis. Phosphorylation of SHP at Thr-58 (Thr-55 in human SHP) by PKCζ likely acts upstream of the RanBP2-mediated SUMOylation of SHP.

This study reveals an unexpected function of a nucleoporin, RanBP2, in regulation of BA levels through SUMOylation of SHP, a key transcriptional regulator in maintenance of BA homoeostasis. When hepatic BA levels are elevated, SHP interaction with RanBP2, a nucleoporin and E3 SUMO ligase, was increased and RanBP2-mediated SUMO2 modification of SHP at K68 (K65 in human SHP) was required for nuclear transport, interaction of SHP with repressive histone modifiers, LSD1 and HDAC1, and the gene repression function of SHP. BA signal-induced phosphorylation of SHP at Thr-58 (Thr-55 in human SHP) by PKCζ is important for increased interaction of SHP with RanBP2 and RanBP2-mediated SUMOylation of SHP (Fig. 9). In previous studies, we concluded that SHP phosphorylation by PKCζ appears to occur in the nucleus based on the observations that both phosphor-SHP and phosphor-PKCζ were detected in the nucleus and at the *Cyp7a1* promoter after CDCA or FGF19 treatment^11^. However, in the previous studies we did not directly determine whether SHP was phosphorylated in the cytoplasm or nucleus. On the basis of the additional information from the present studies that phosphorylation precedes SUMOylation and nuclear transport of SHP, the correct interpretation of the results is that phosphorylation by PKCζ occurs in the cytoplasm and is required for interaction of SHP with RanBP2.

An intriguing finding is the identification of a component of the nucleopore filaments and one of few identified E3 SUMO ligases, RanBP2, as a SUMO ligase for SHP. Previously, nuclear import was shown to correlate with SUMOylation of nuclear proteins^34^ suggesting that RanBP2-mediated SUMOylation and transport of SHP are likely linked. We show that SUMO2 modification of SHP by RanBP2 is required for nuclear localization of SHP and interaction with co-repressors, LSD1 and HDAC1, and thereby, is important for the feedback transcriptional repression of BA synthesis and import genes by SHP in response to elevated hepatic BA levels under physiological conditions. RanBP2-mediated SUMOylation is relatively specific for SHP, since SUMOylation of two other nuclear receptors, FXR and LRH-1, which are also important for regulation of BA levels and are targets of SUMO modification (28–30), is not dependent on RanBP2. It remains possible, however, that RanBP2 mediates SUMOylation of other transcriptional regulators.

In both ANIT and chronic BA feeding experiments in mice, we observed liver toxicity and hepatic inflammation was exacerbated by expression of the SUMO-defective K68R mutant compared with WT SHP, suggesting that RanBP2-mediated SUMOylation of SHP plays a critical role in both normal and pathological conditions by protecting the liver from excessively elevated BA levels. FGF15 is an intestinal hormone that strongly represses BA synthetic genes in a SHP-dependent manner^30^. Notably, in physiological conditions without a biliary insult, mice expressing the K68R SHP mutant had decreased levels of an FXR antagonist, TβMCA (ref. 29), in the intestine, and dramatically increased expression of intestinal *Fgf15* gene. In these mice, even though intestinal *Fgf15* expression was increased, expression of *Cyp7a1* and *Cyp8b1* genes was upregulated. Furthermore, FGF19 or CDCA inhibition of expression of these genes was markedly blunted in SHP-KO hepatocytes expressing the K68R-SHP mutant, suggesting a critical role of SUMO2 modification of SHP in mediating inhibition of BA synthetic genes in response to both BA and FGF19 signalling.

In the present study, we also observed that both *Cyp7a1* and *Cyp8b1* mRNA levels were increased in mice expressing the K68R-SHP mutant in normal physiological conditions, but these two genes were differentially regulated upon ANIT or chronic CA feeding. The mechanism for the differential regulation is not clear but the stress-signalling pathway upon biliary insults may play a role in the selective repression of *Cyp7a1*. It has been shown that *Cyp7a1* regulation is influenced by multiple factors, including stress signalling, nutrient status, diurnal regulation and hepatic inflammation, in addition to BA and cholesterol levels^2^,^35^. We also observed that expression of *Cyp8b1* is highly elevated in ANIT-treatment mice. MAFG was recently identified as a global transcriptional repressor of BA synthesis and metabolism^36^. Upon induction by BA-activated FXR, MAFG directly binds to the *Cyp8b1* promoter and represses expression of this gene, but MAFG does not directly repress *Cyp7a1* (ref. 36). In the present study, we observed that expression of *Mafg* was significantly downregulated in mice expressing the SHP-K68R mutant upon biliary insults, either ANIT treatment or BA overload (Supplementary Fig. 13), which may contribute to the increased expression of *Cyp8b1*.

SUMOylation is a highly dynamic protein modification that modulates diverse cellular processes, including transcription, DNA repair, genome stability and intracellular trafficking^15^,^18^. The SUMOylation of lipid-sensing nuclear receptors, such as PPARγ, LRH-1, LXR and FXR, mediated gene-selective repression of inflammatory responses^26^,^28^,^37^. SUMO2 modification of FXR selectively repressed NF-κB target inflammatory genes and the anti-inflammatory function by SUMOylated FXR was impaired by elevated acetylation of FXR in obesity^26^. Remarkably, a recent study has demonstrated that mutation of a single Lys residue, K289, which blocks SUMOylation of LRH-1 is sufficient to protect the mice against atherosclerosis by upregulating liver reverse cholesterol transport genes^27^. The present study that SUMOylation facilitates nuclear transport of SHP, as well as, increases SHP interaction with repressive histone-modifying enzymes, adds to the diverse functional consequences of SUMOylation of nuclear receptors.

In conclusion, we show that SUMO2 modification of SHP at a single Lys residue, K68, is important for nuclear localization of SHP and its interaction with LSD1 and HDAC1, which results in feedback transcriptional responses that maintain BA homoeostasis and protect the liver from bile toxicity. Further, the current study identifies SHP as the first nuclear receptor that is a direct target of SUMOylation by RanBP2. RanBP2-mediated SUMOylation of SHP may be an effective therapeutic target for the treatment of cholestatic liver diseases and other BA-related hepatobiliary diseases.

## Methods

### Animal experiments

Eight-week-old BALB/c male mice were fasted for at least 6 h and then fed chow supplemented with 0.5% CA (CA chow) acutely for 3 h from 1700 to 2000 hours before being killed for acute feeding experiments. Adenovirus expressing GFP, SHP WT or K68R mutant (2.5–5.0 × 10^8^ active viral particles in 100 μl saline) was injected via the tail vein and 2 weeks later, serum and tissues were collected. Twelve-week-old SHP-KO male mice were infected by adenovirus expressing GFP, SHP WT or K68R mutant and 2 weeks later, serum and tissues were collected. Lentivirus (0.5–1.0 × 10^9^ IU per ml in 100 μl saline) was injected via the tail vein and after one month, mice were treated by gavage with vehicle (olive oil) or 75 mg kg^−1^ ANIT for 48 h as described^31^ or were fed a CA chow for 5 days for chronic feeding experiments. All animal use and viral protocols were approved by the University of Illinois, Urbana-Champaign Institutional Animal Care and Biosafety Committees (IACUC).

### Liver histology and toxicity

For IHC studies, paraffin-embedded liver sections were incubated with F4/80 antibody overnight at 4 °C and detected with the rabbit-specific HRP/DAB Detection IHC Kit (Abcam). The sections were stained with haematoxylin and imaged with a NanoZoomer Scanner (Hamamatsu). Serum ALT/AST levels, BA levels and total bilirubin levels were measured using colorimetric analysis kits purchased from Sigma, Trinity Biotech and Biovision, respectively. BA composition in the liver and intestine was detected by liquid chromatography–mass spectrometry analysis (AB Sciex, Foster City, CA).

### Nuclear localization studies

For IF studies, cells were fixed with 4% paraformaldehyde, permeabilized with PBS containing 3% BSA, 0.1% Triton X-100 (PBS-BT), incubated with SHP (H-160) or M2 and RanBP2 (D-4) antibody for 2 h, washed and incubated with Alexa Fluor 488-conjugated donkey anti-mouse or -rabbit IgG and Alexa Fluor 647-conjugated donkey anti-rabbit or -mouse IgG for 1 h as indicated. Nuclei were stained with Hoechst 33,258 and the samples were scanned with an LSM 700 (Carl Zeiss). SHP (H-160) and RanBP2 (D-4) antibodies for IF were purchased from Santa Cruz Biotech. For IHC studies, paraffin-embedded liver sections were incubated with SHP (H-160) antibody overnight at 4 °C and detected with the rabbit-specific HRP/DAB Detection IHC Kit (Abcam). The sections were stained with haematoxylin and eosin (H&E) and imaged with a NanoZoomer Scanner (Hamamatsu).

### SUMO assays

The *in vitro* SUMO assay was performed as described^26^ using purified SUMO components^38^. Reaction mixtures containing 500 ng SUMO2 protein, 100 ng E1 (SAE1/SAE2), 200 ng E2 (Ubc9) and flag-SHP were incubated at 30 °C for 1 h. SUMOylated proteins were detected by IB. For in-cell assays, transfected HepG2 cells were treated with 50 μM CDCA for 15 min, lysed in IP buffer (50 mM Tris-HCl, pH 8.0, 150 mM NaCl, 1 mM EDTA, 0.5% NP-40, 5% glycerol, 0.1% SDS, protease inhibitors and 20 mM NEM), immunoprecipitated with SHP antibody and SUMOylated proteins were analysed by IB. For *in vivo* assays, flag-SHP or endogenous SHP in mouse liver was immunoprecipitated at 4 °C for 3 h in IP buffer containing 20 mM NEM and SUMO-SHP was detected by IB. Uncropped scans of all of the immunoblots are shown in Supplementary Fig. 14.

### Molecular and biochemical assays

HepG2 cells were co-transfected with 200 ng luciferase reporter plasmids, 300 ng of CMV β-galactosidase plasmid and 10–50 ng of expression plasmids for flag-SHP WT or mutant. Luciferase activities were normalized to β-galactosidase activities. Primary mouse hepatocytes, prepared as described^26^, were infected with Ad-SHP WT or mutant, treated with CDCA for 1 h, and then, ChIP assays were performed. The GST pull-down, CoIP and ChIP assays were performed as described previously^26^,^39^. Primer sequences for ChIP quantitative PCR are shown in Supplementary Table 1.

### Quantification of mRNA

RNA was isolated from liver or cultured cells and quantified by quantitative real-time PCR, normalized to 36B4 mRNA. Primer sequences for quantitative real-time PCR are shown in Supplementary Table 1.

### Data availability

The data that support the findings of this study are available from the corresponding author upon request

## Additional information

**How to cite this article:** Kim, D.-H. *et al.* Critical role of RanBP2-mediated SUMOylation of Small Heterodimer Partner in maintaining bile acid homoeostasis. *Nat. Commun.* 7:12179 doi: 10.1038/ncomms12179 (2016).

## Supplementary Material

Supplementary Information Supplementary Figures 1-14, Supplementary Table 1, Supplementary Methods and Supplementary References
