# Supplementary material for: Critical role of RanBP2-mediated SUMOylation of Small Heterodimer Partner in maintaining bile acid homeostasis
Source: Nat Commun. 2016 Jul 14;7:12179. doi: 10.1038/ncomms12179 (PMC4947186; doi:10.1038/ncomms12179)
Supplement: Supplementary Information — Supplementary Figures 1-14, Supplementary Table 1, Supplementary Methods and Supplementary References [file ncomms12179-s1.pdf]

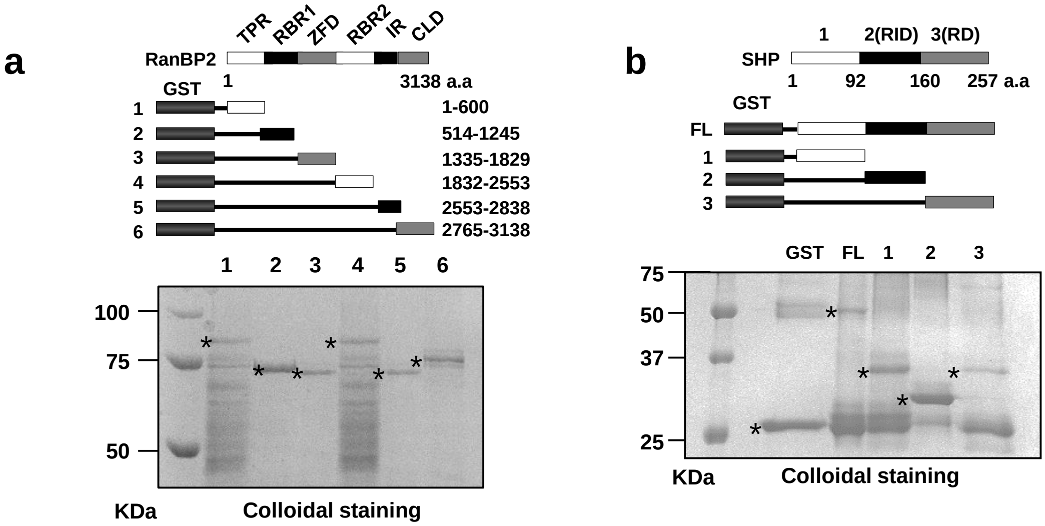

Supplementary Figure 1. GST-RanBP2 and GST-SHP fusion proteins used in GST pull-down assay.
Schematics of the RanBP2 (a) and SHP (b) domains fused to GST are shown at the top. GST-RanBP2 and GST-SHP fusion proteins used in GST pull down interaction studies were visualized by Colloidal staining.

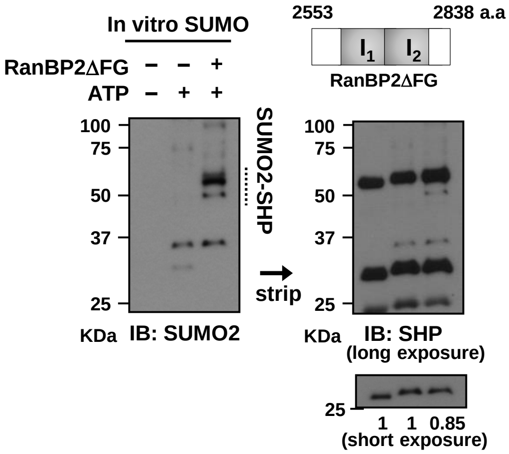

Supplementary Figure 2. RanBP2 SUMOylates SHP in vitro.
Purified SUMO2, E1 enzyme, Ubc9, and flag-SHP immunoprecipitated from Cos-1 cells were incubated as indicated with ATP and a RanBP2 fragment (RanBP2 ΔFG) that retains E3 SUMO catalytic activity. SUMOylated SHP was detected by IB with SUMO2 antibody.

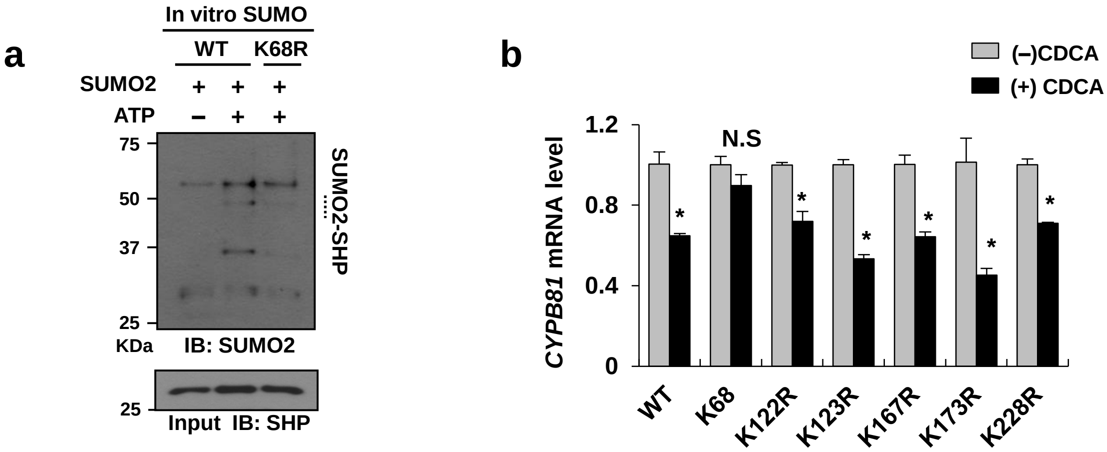

Supplementary Figure 3. Effects of the lysine mutations of SHP on SUMOylation in vitro and on inhibition of the BA biosynthetic gene, *CYP8B1*.

a. Immunoprecipitated SHP or the K68R mutant was incubated with SUMO components and RanBP2 ΔFG as in (Supplementary Figure 2) and SUMOylated SHP was detected by IB with antibody to SUMO2.

b. Effects of Lys mutations of SHP on expression of a key BA biosynthetic gene, *CYP8B1.* Flag-SHP or the indicated mutants were expressed in HepG2 cells, and then the cells were treated with vehicle or 50 μM CDCA for 6 h, and levels of *CYP8B1* mRNA were determined by qRT-PCR. The control samples treated with vehicle are set to 1 for each SHP protein. Statistical significance was determined by the Student's t-test (SEM, n=3, *, $p<0.05$, NS, not significant).

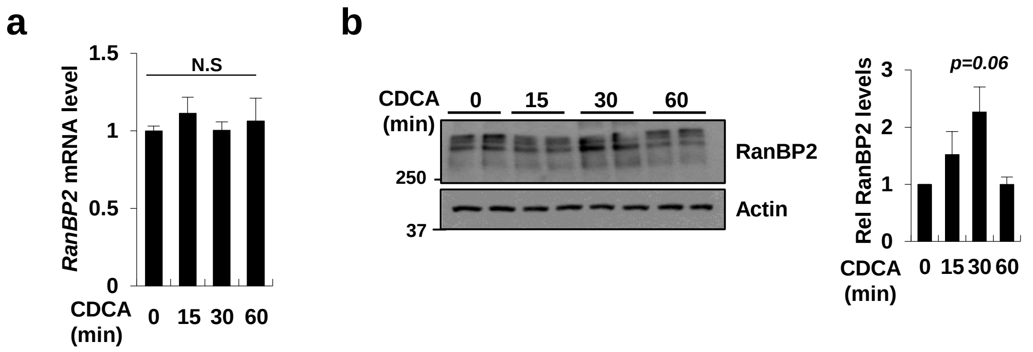

Supplementary Figure 4. Effects of CDCA on RanBP2 expression.

a. Hepa1c1c7 cells were treated with vehicle or 50 μM CDCA for the indicated times, and levels of RanBP2 mRNA were determined by qRT-PCR. The control samples treated with vehicle are set to 1. Statistical significance was determined by the Student's t-test (SEM, n=3, *, $p<0.05$, NS, not significant).

b. RanBP2 after treatment of Hepa1c1c7 cells with CDCA for the indicated times was detected by IB. At right, the intensities of the bands were quantified and values for control (0 min) group are set to 1

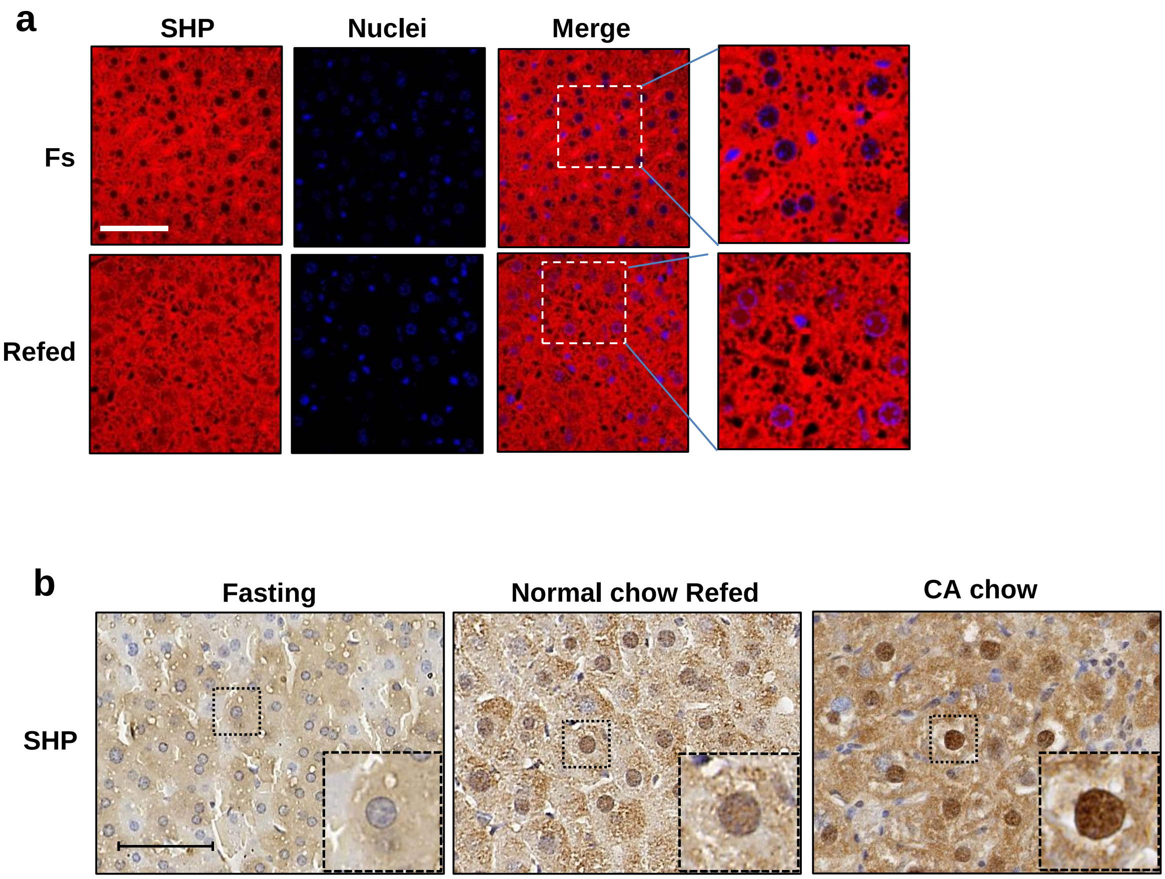

Supplementary Figure 5. Effects of feeding or CA feeding on nuclear localization of SHP in mouse liver.

a. Endogenous SHP in livers of mice fasted or refed normal chow for 3 h were detected by IF as described in the Methods. Scale bar, 50μm.

b. Endogenous SHP in livers of mice fasted, refed normal chow or CA chow (0.5%) for 3 h were detected by IHC as described in the Methods. Scale bar, 50μm.

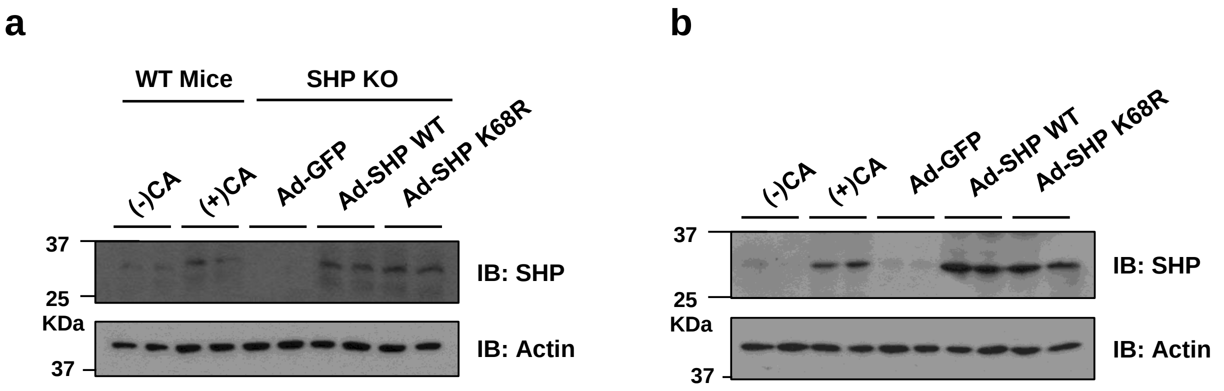

Supplementary Figure 6. Adenoviral injection resulted in hepatic expression of flag-SHP WT and K68R mutant at levels similar to the induced SHP levels in BA-fed mice.

Mice were acutely fed normal chow or BA (0.5 % CA)-supplemented chow for 6 h and liver extracts were prepared. For adenoviral experiments, SHP-KO (a) or wild-type (b) mice were tail vein injected with adenovirus expressing SHP (WT), the K68R mutant, or control Ad-GFP for 2 weeks and liver extracts were prepared. Hepatic SHP protein levels were detected by IB using SHP antibody.

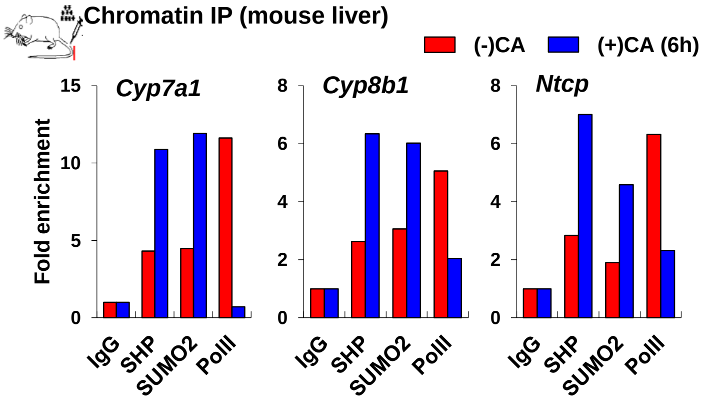

Supplementary Figure 7. Effects of CA feeding on occupancy of SHP and SUMO2. Mice were fed normal chow or BA (0.5% CA)-supplemented chow for 6 h; livers from 2 mice were pooled; chromatin was isolated and precipitated with IgG or antibody as indicated; and the enrichment of DNA sequence for *Cyp7a1, Cyp8b1 or Ntcp* was determined.

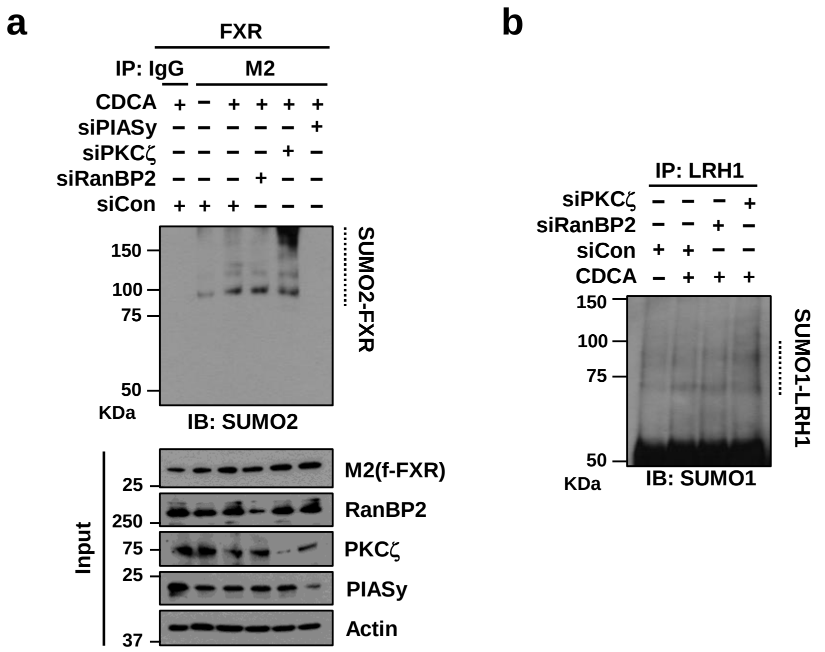

Supplementary Figure 8. Effects of downregulation RanBP2, PKCζ, and PIASy on FXR or LRH1 SUMOylation.

HepG2 cells were transfected with expression plasmids for flag-FXR or LRH-1 and with siRNA for PKCζ or an E3 SUMO ligase, either RanBP2 or PIASy, as indicated, and then, the cells were treated with 50 μM CDCA for 15 min. SUMOylated FXR (a) or LRH-1 (b) were detected by the IP/IB. Protein levels in input samples were detected by IB.

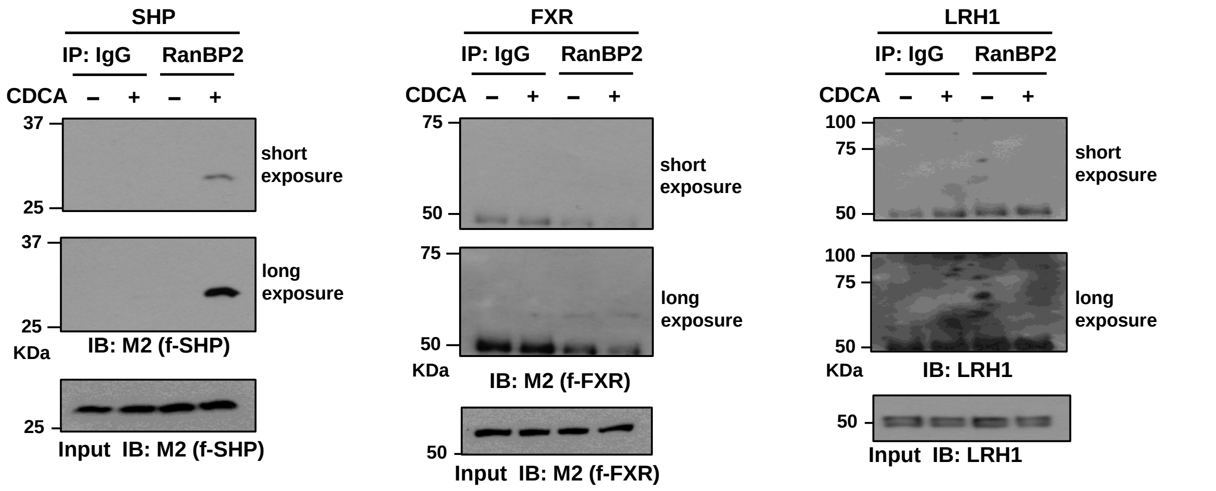

Supplementary Figure 9. Effects of CDCA treatment on RanBP2 interaction with SHP, FXR, or LRH-1.
HepG2 cells were transfected with expression plasmids for flag-SHP, flag-FXR or LRH-1 as indicated, and then, the cells were treated with 50 μM CDCA for 30 min. Nuclear receptor interaction with RanBP2 was detected by CoIP using M2 antibody for SHP and FXR and LRH-1 antibody for LRH-1. Protein levels in input samples were detected by IB.

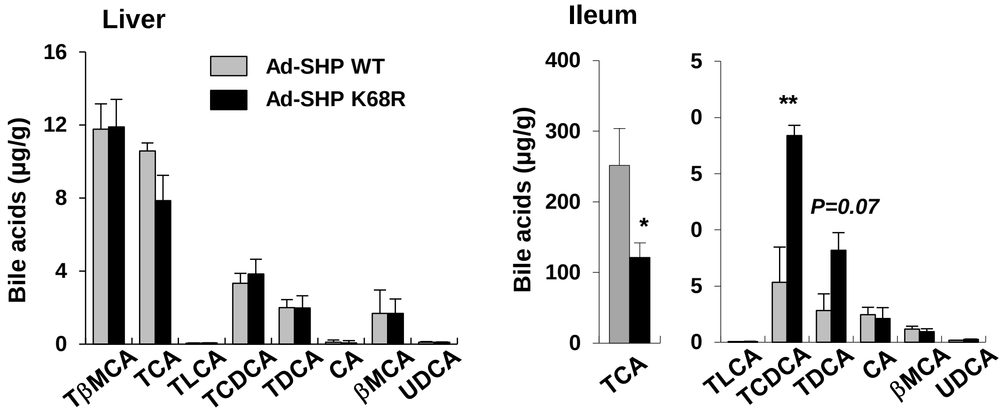

Supplementary Figure 10. Effects of adenoviral-mediated expression of the K68R mutant in the liver and small intestine on bile acid composition. Mice (n=5) were tail vein injected with adenovirus expressing SHP (WT) or the K68R mutant for 2 weeks and liver and the ileum were removed. The composition of BAs in extracts of the liver and ileum were determined by LC/MS/MS as described in Methods. Statistical significance was determined by the Student's t-test, (SEM, n=5 mice, *p<0.05, **p<0.005).

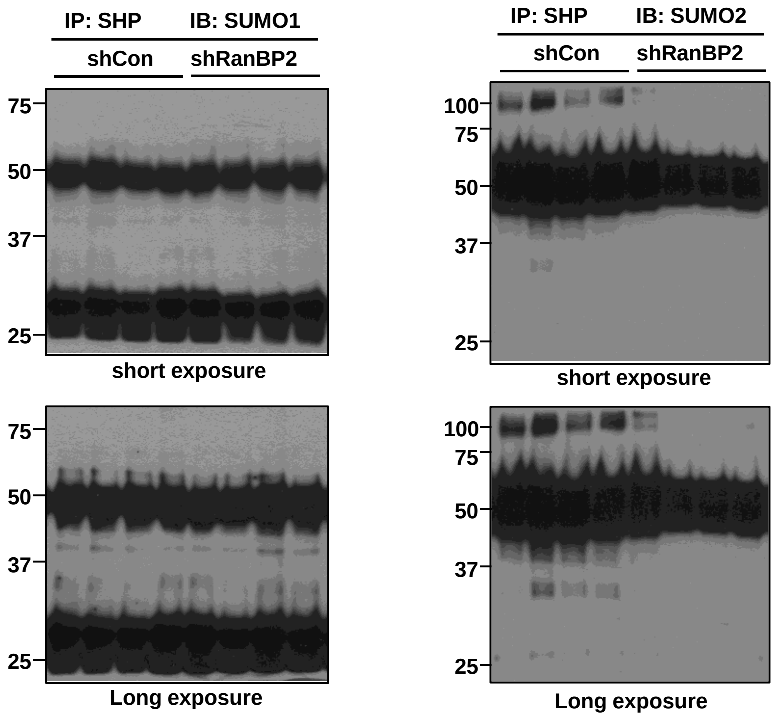

Supplementary Figure 11. Effect of downregulation of RanBP2 on SUMO1 or SUMO2 modification of SHP in mice.

In vivo SUMO assay: SUMO-SHP levels in RanBP2-downregulated mice liver were detected by IP/IB. Endogenous SHP was immunoprecipitated by SHP antibody and detected by SUMO1 or SUMO2 antibody.

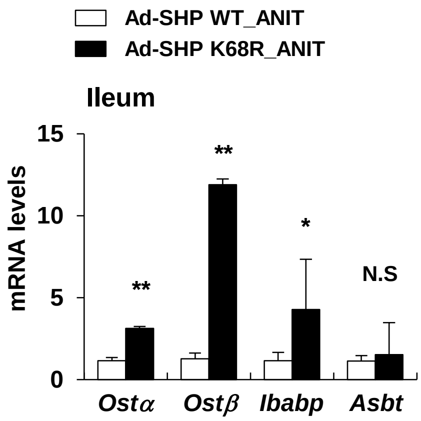

Supplementary Figure 12. Effects of adenoviral-mediated expression of the SHP K68R mutant on gene expression in the ileum.

Expression of indicated genes in the ileum was measured by q-RTPCR. The values for WT SHP are set to 1. Statistical significance was determined by the Student's t-test, (SEM, n=5 mice, *p<0.05, **p<0.005, and NS, statistically not significant).

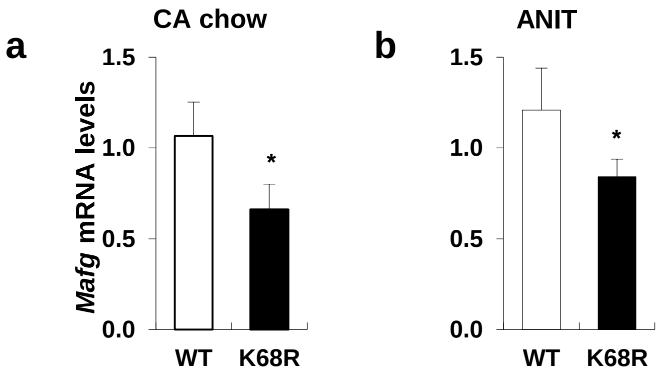

Supplementary Figure 13. Effects of adenoviral-mediated expression of the SHP K68R mutant in liver on *Mafg* expression in mice challenged with BA overload or treatment with ANIT.

Levels of hepatic *Mafg* mRNA in mice fed 0.5% CA-supplemented chow chow for 5 days (a) or treated with ANIT (b) was measured by q-RTPCR. The values for WT SHP are set to 1. Statistical significance was determined by the Student's t-test, (SEM, n=5 mice, *p<0.05, and NS, statistically not significant).

Supplementary Figure 14. Uncropped blots

Figure 1b

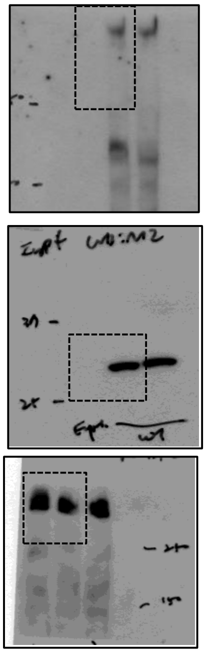

Figure 1c

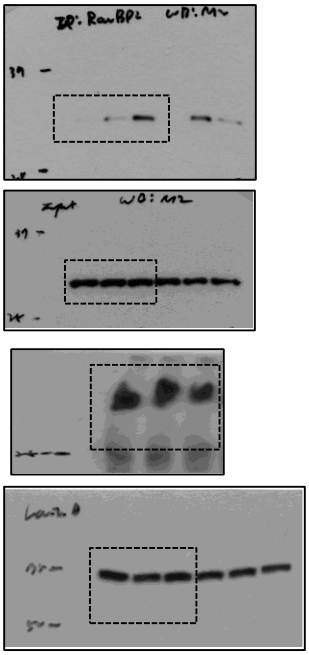

Figure 1d

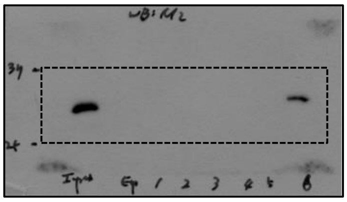

Figure 1e

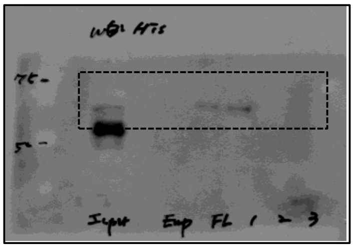

Figure 2a

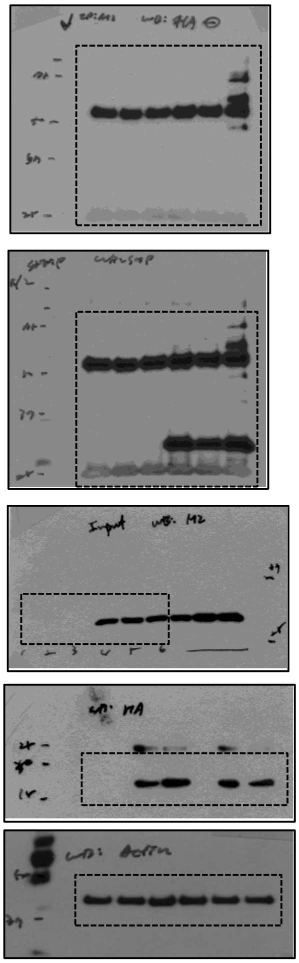

Figure 2b

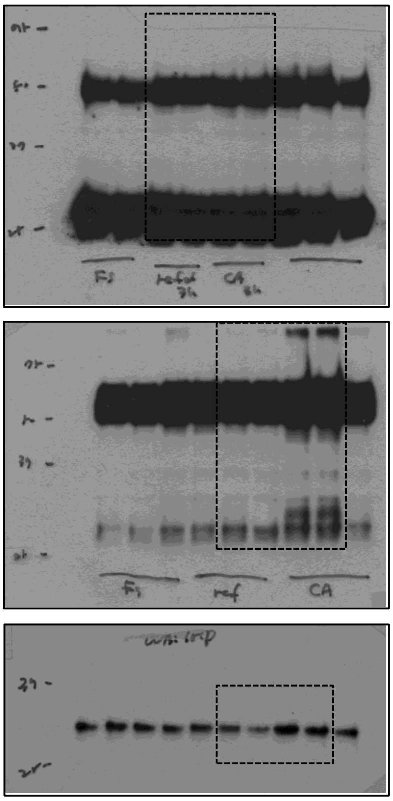

Figure 2c

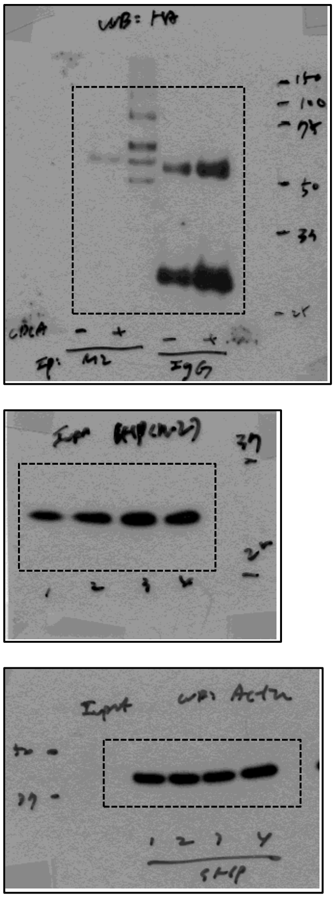

Figure 2d

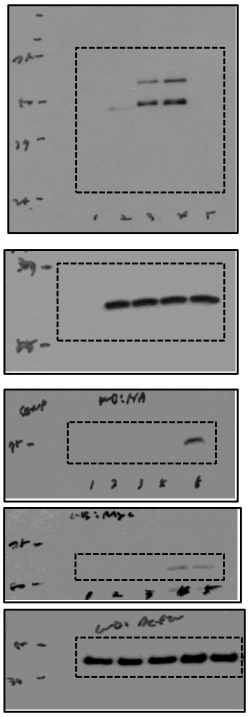

Figure 2e

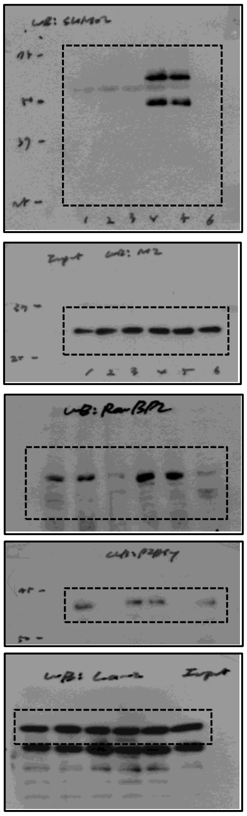

Figure 2f

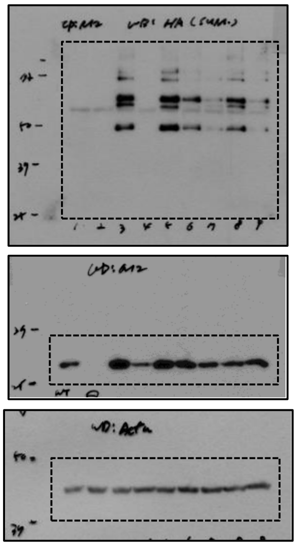

Figure 3c

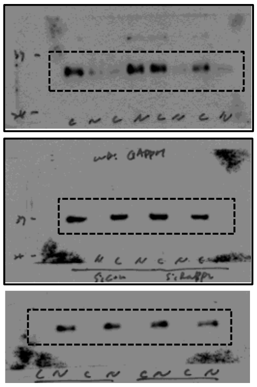

Figure 3c

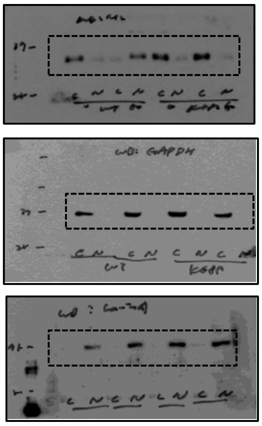

Figure 4b

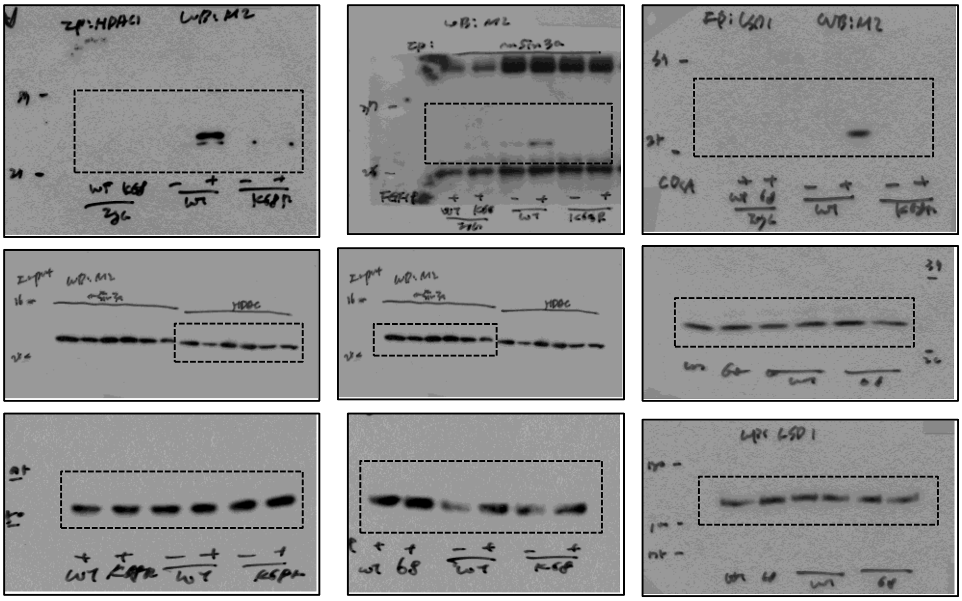

Figure 5a

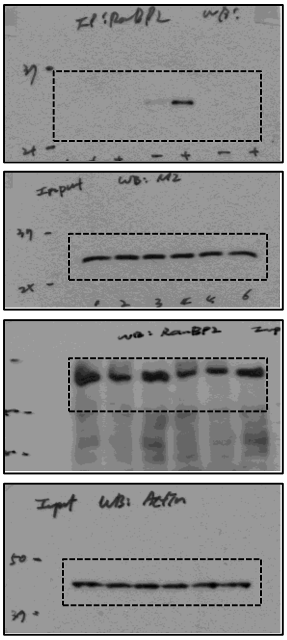

Figure 5b

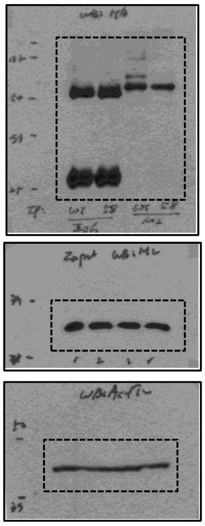

Figure 5c

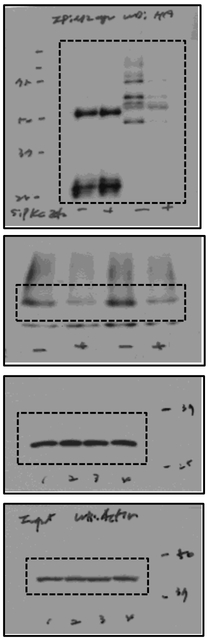

Figure 5d

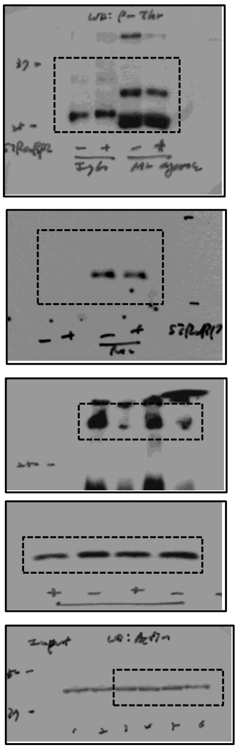

Figure 6a

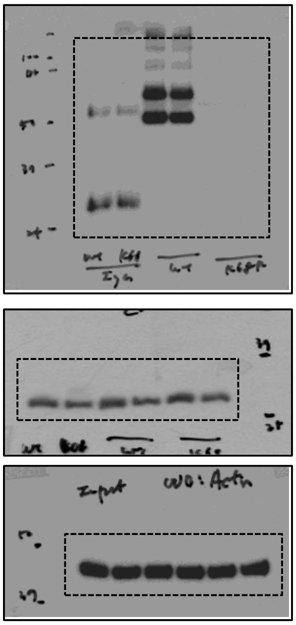

Figure 7b

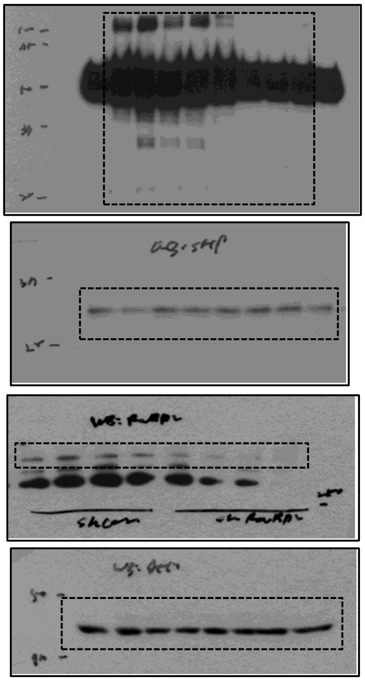

Figure 7f

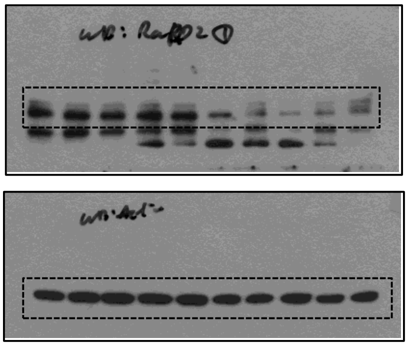

Supplementary Table 1

Primers used in this study (ChIP and q-RTPCR)

	Purpose	Definition	Sense Primer	Anti-sense Primer
human	q-RTPCR	36B4	TGCTGAACATGCTCAAC	GTCGAACACCTGCTGGATGAC
human	q-RTPCR	CYP7A1	TGGGCATCGCAAGCAAA	CTTTCATTGCTTCTGGGTTCCTA
human	q-RTPCR	CYP8B1	TTCGCTTCTGCTATTACATCTT	TCCTGCTCCTTGTCCTTC
mouse	ChIP	Cyp7a1	ATATGCACAGGACCATGATC	CTTTGGTAGGTGAGCTCTTC
mouse	ChIP	Cyp8b1	AAGCATGGGGATGTGTTCAC	CAAACTTGCGGAACTCCATG
mouse	q-RTPCR	36B4	CCCTGAAGTGCTCGACATCA	TGCGGACACCCTCCAGAA
mouse	q-RTPCR	RanBP2	ACTGGCGAGGAAGATGAAGA	CGTTCCTTCCATTCTTTGGA
mouse	q-RTPCR	Cyp27a1	GACAACCTCCTTTGGGACTTAC	GTGGTCTCTTATTGGGTACTTGC
mouse	q-RTPCR	Cyp7a1	AACGGGTTGATTCCATACCTGG	GTGGACATATTTCCCCATCAGTT
mouse	q-RTPCR	Cyp7b1	GACGATCCTGAAATAGGAGCACA	AATGGTGTTTGCTAGAGAGGCC
mouse	q-RTPCR	Cyp8b1	GAATCTAACCAGGCCATGCT	AGGAGCTGGCACCTAGACT
mouse	q-RTPCR	Bsep	CAATGTTCAGTTCCTCCGTTCA	TTTGGTGTTGTCCCCSTSCTTG
mouse	q-RTPCR	Mrp2	TATCCCCGGGAAATCTGTTC	TAACCAACATTCTCCGCGC
mouse	q-RTPCR	Oatp1	GTCTTACGAGTGTGCTCCAGAT	GGAATACTGCCTCTGAAGTGGATT
mouse	q-RTPCR	Oatp2	GACGGCTCAGTGTTCATTC	CTTCTAGCTGGTCCCTCTT
mouse	q-RTPCR	Oatp4	GATCCTTCACTTACCTGTTCAA	CCTAAAAACATTCCACTTGCCATA
mouse	q-RTPCR	Ntcp	TACCTCCTCCCTGATGCCTTTC	TGCGTCTGCAGCTTGGATTTA
mouse	q-RTPCR	Fas	CCTGGATAGCATTCCGAACCT	AGCACATCTCGAAGGCTACACA
mouse	q-RTPCR	Cyt-c	GGAGGCAAGCATAAGACTGG	TCCATCAGGGTATCCTCTCC
mouse	q-RTPCR	Mcad	GATCGCAATGGGTGCTTTTGATAGAA	AGTTGATTGGCAATGTCTCCAGCAAA
mouse	q-RTPCR	Cpt-1	CAGCATTCTTCGTGACGTTGG	TCGAAACATCTACCATGCAGCA
mouse	q-RTPCR	Srb1	TGCTGTGGTTCGAACAGAGC	GCCTGAATGGCCTCCTTATC
mouse	q-RTPCR	Hmgcr	CTTGTGGAATGCCTTGTGAATG	AGCCGAAGCAGCACATGAT
mouse	q-RTPCR	Srebp1c	GCTGTTGGCATCCTGCTATC	TAGCTGGAAGTGACGGTGGT
mouse	q-RTPCR	Cxcl2	TCCAGAGCTTGAGTGTGACG	TTCAGGGTCAAGGCAAACTT
mouse	q-RTPCR	Tnfα	AGCCCCCAGTCTGTATCCTT	GGTCACTGTCCCAGCATCTT
mouse	q-RTPCR	IL8	TCACCGATGTCTACCTGCTG	CACAGGGTTGAGCCAAAAGT
mouse	q-RTPCR	Fgf15	TTCAGGGAGGAAATGGACTG	TGGTCCTGGAGCTGTTCTCT
mouse	q-RTPCR	Ibabp	TCATCACAGAGGTCCAGCAG	CACATTCTTTGCCAATGGTG
mouse	q-RTPCR	Ostα	TGGACCCTGGAAGACATA	TAACCACTGATAAGGCTGAG
mouse	q-RTPCR	Ostβ	ATCTTGATGACTCCATAATG	GTCTTTCTCTTTCAACTCA
mouse	q-RTPCR	Mafg	GACCCCCAATAAAGGAAACAA	TCAACTCTCGCACCGACAT
mouse	q-RTPCR	Asbt	TGGAATGCAGAACACTCAGC	GCAAAGACGAGCTGGAAAAC

Supplementary Table 1. Lists of primer sequences for ChIP q-PCR and q-RTPCR studies.

Supplementary Methods

Material Information. Antibodies for SHP(H-160, 1:3000), SUMO1(D-11, 1:3000), HDAC1(H-51, 1:3000), LRH1(L-15, 1:3000), FXR(H-130, 1:3000), mSin3a(K-20, 1:3000), HNF4 alpha(H-171, 1:3000), RanBP2(D-4, 1:2500), HA(Y-11, 1:5000) were purchased from Santa Cruz Biotech; and for SUMO2(4917S, 1:3000), PKCζ(9368S, 1:3000), PIASy(4392S, 1:3000), LSD1(2139S, 1:3000) from Cell Signaling. RanBP2(ab2938, ab64276, 1:2500) was obtained from Abcam. F4/80(AP10243PU-M, 1:200) was obtained from Acris Antibodies, Inc. Alexa Flour 488-conjugated donkey anti-mouse IgG(A21202, 1:500), Alexa Fluor 647-conjugated donkey anti-rabbit IgG(A21245, 1:500), were purchased from Thermo Fisher Scientific, Inc. M2 antibody(F3165, 1:15000), M2 agarose(A2220), Hoechst 33258(14530), and RanBP2 lentiviral vector(TRCN0000306503) were purchased from Sigma, Inc. Bile acids, TCDCA, TDCA, CA, UDCA, DCA, CDCA, and LCA, were purchased from Sigma Inc, TβMCA and TLCA from Santa Cruz Biotech, and βMCA from TRC, Inc. ON-TARGETplus siRNAs for mouse RanBP2 (L-042297-01, SMART pool, GUAGAAUGUUAUAAGCGUU, UCAUGUAAAUAUCCGACUA, AAUUAAACCCAACGCAAAA, GUACUCUGAUUCACCGAAA); for human RanBP2 (L-004746-00, SMART pool, GCGAAGUGAUGAUAUGUUU, CAAACCACGUUAUUACUAA, CAGAACAACUUGCUAUUAG, GAAGGAAUGUUCAUCAGGA); and mouse PIASy (L-048649-01, SMART pool, AGAGUGGGCUGAAGCACGA, AGGUGGAGAUGAUCCGCAA, CUUUGUACCUGGUGCGACA, GUGAUGAGCUUCCGAGUAU) were purchased from GE Healthcare Dharmacon, Inc. and Silencer Select PKCζ siRNA was used as previously[1] and was purchased from Ambion (4390825, CGUUCGACAUCAUCACCGAtt)

Adenoviral and lentiviral vector constructions: The SUMO-defective flag-mouse SHP mutant was constructed by site-directed mutagenesis (Stratagene, Inc.) of Ad-flag-mouse SHP. Adenoviral vectors were prepared as previously described[2]. Lentivirus packaging plasmids pMD2.G and psPAX2 (Addgene) were used to make Lenti-Empty and Lenti-shRanBP2.

Construction of GST-RanBP2 constructs: Sequence encoding the RanBP2 fragments (Fig. 1d) was amplified by PCR, and the PCR product was inserted into the pGEX4T-1 vector (GE Healthcare) at Sal1 and Not1 sites.

Supplementary References

1. Seok, S., Kanamaluru, D., Xiao, Z., Ryerson, D., Choi, S. E., Suino-Powell, K., Xu, H. E., Veenstra, T. D. & Kemper, J. K. Bile acid signal-induced phosphorylation of small heterodimer partner by protein kinase Czeta is critical for epigenomic regulation of liver metabolic genes. *J. Biol. Chem.* **288**,23252-23263 (2013)

2. Fang, S., Miao, J., Xiang, L., Ponugoti, B., Treuter, E. & Kemper, J. K. Coordinated recruitment of histone methyltransferase G9a and other chromatin-modifying enzymes in SHP-mediated regulation of hepatic bile acid metabolism. *Mol. Cell. Biol.* **27**,1407-1424 (2007)
